# Synthetic Approaches to Piperazine-Containing Drugs Approved by FDA in the Period of 2011–2023 †

**DOI:** 10.3390/molecules29010068

**Published:** 2023-12-21

**Authors:** Maria Novella Romanelli, Laura Braconi, Alessio Gabellini, Dina Manetti, Giambattista Marotta, Elisabetta Teodori

**Affiliations:** Section of Pharmaceutical and Nutraceutical Science, Department of Neurosciences, Psychology, Drug Research and Child Health (NEUROFARBA), University of Florence, Via Ugo Schiff, 6, Sesto Fiorentino, 50019 Florence, Italy; laura.braconi@unifi.it (L.B.); alessio.gabellini@unifi.it (A.G.); dina.manetti@unifi.it (D.M.); giambattista.marotta@unifi.it (G.M.); elisabetta.teodori@unifi.it (E.T.)

**Keywords:** kinase inhibitors, receptor modulators, Buchwald–Hartwig amination, aromatic nucleophilic substitution, reductive amination, Finkelstein alkylation, amide bond formation

## Abstract

The piperazine moiety is often found in drugs or in bioactive molecules. This widespread presence is due to different possible roles depending on the position in the molecule and on the therapeutic class, but it also depends on the chemical reactivity of piperazine-based synthons, which facilitate its insertion into the molecule. In this paper, we take into consideration the piperazine-containing drugs approved by the Food and Drug Administration between January 2011 and June 2023, and the synthetic methodologies used to prepare the compounds in the discovery and process chemistry are reviewed.

## 1. Introduction

Piperazine is among the most frequently used heterocycles in biologically active compounds [1,2,3]. This moiety is useful for different reasons: for its impact on the physicochemical properties of the final molecule, for its structural and conformational characteristics and for its easy handling in synthetic chemistry. Indeed, many surveys can be found in the literature on the application of the piperazine ring in biologically active compounds within different research areas (see [4] and the references cited therein).

In a previous paper, we discussed the role of the piperazine ring, first analyzing the drugs approved by the Food and Drug Administration (FDA) since 2017 that showed such a moiety, and then looking at biologically active piperazine derivatives for specific therapeutic areas [4]. As described there, the piperazine moiety is mainly used as a basic and hydrophilic group to optimize the pharmacokinetic properties of the final molecule or as a scaffold to arrange pharmacophoric groups in the proper position in the interaction with the target macromolecules. Our long-lasting interest in this field prompted us to also revise the synthetic procedures that have been mostly used in medicinal and process chemistry to prepare piperazine-containing drugs. It must be noticed that, owing to the popularity of this moiety, many useful synthons are commercially available: either (a) *N*-acyl or *N*-aryl piperazines decorated with protecting groups and/or with functional groups useful for further expansion of the molecule, or (b) piperazines carrying substituents such as phenyl, methyl or carboxylic acid, among others, on the ring *C*-atom.

The synthetic procedures developed to build the piperazine ring or to insert substituents have been the topic of some reviews [5,6,7,8]; such methods allow obtaining piperazine derivatives with a high degree of substitution on the ring. However, the structural complexity of the piperazine moiety in biologically active molecules varies considerably. We analyzed the structures of FDA-approved drugs from the period from January 2011 to June 2023 (Table 1 and Table 2); the compounds were divided according to the complexity around the piperazine ring. Table 1 shows molecules having substituents on one (**1**–**3**) or both piperazine nitrogen atoms (**4**–**28**), and they are listed according to the kind of substitution, grouped into monoaryl (**1**–**3**), diaryl (**4**, **5**), aryl-alkyl (**6**–**17**), dialkyl (**18**–**24**), alkyl-acyl (**25**, **26**) and diacyl piperazines (**27**, **28**).

Table 2 reports a smaller number of molecules, with higher complexity on the piperazine ring. In these compounds, substituents are present on one or more *C*-atoms. In compound **29**, the piperazine ring is inserted into a 3,6-diazabicyclo[3.1.1]heptane ring. One *C*-position of the piperazine moiety is substituted in compounds **30**–**32**, and in **33**–**40**, the piperazine ring is included in a more complex polycyclic structure. An endocyclic carbonyl function characterizes compounds **38**–**40**.

An analysis of the synthetic methods used to prepare these drugs is reported in Section 2 and Section 3, with emphasis on the chemistry involving the assembly, decoration and reactivity of piperazine or of the building block containing it. Our analysis is mainly limited to the synthetic methods applied in the medicinal chemistry and in the process chemistry routes developed by the originator company; procedures developed by other researchers or by generic industries are taken into account only in a few instances.

## 2. Synthesis of Drugs Carrying a Piperazine Ring Decorated Only on *N*-Atoms

### 2.1. N-Aryl Derivatives

The main synthetic methods to obtain *N*-arylpiperazine from aromatic compounds (often halides) and piperazine are Pd-catalyzed Buchwald–Hartwig coupling, the Cu-catalyzed Ullmann–Goldberg reaction and aromatic nucleophilic substitution (SN_Ar_) on electron-deficient (hetero)arenes [9,10]. Alternatively, the piperazine ring can be built from a suitable aniline and bis-(2-haloethyl)amine or diethanolamine. These methods have been widely used in the discovery chemistry, but not all may be suitable in the synthetic procedures applied for the clinical or commercial supply, owing to possible problems in the scale-up process regarding yield, purification or safety concerns.

Palbociclib (**1**), Ribociclib (**2**), Trilaciclib (**6**) and Abemaciclib (**21**) are Cyclin-Dependent Kinase (CDK) 4/6 inhibitors showing selectivity over CDK 1,2,5,7,9. Compounds **1**, **2** and **21** were approved for the treatment of metastatic breast cancer, and **6** is used to reduce myelosuppression induced by chemotherapy treatments in small cell lung cancer (SCLC). The X-ray structures of **1**, **2** and **21** with CDK6 show that the compounds bind to the kinase-inactive conformation. The 2-aminopirimidine moiety interacts with the hinge region, and the positively charged piperazine ring lies in the solvent-exposed region close to Thr107 and Asp104. The interaction with the latter contributes to CDK4/6 selectivity [11]. The medicinal chemistry and synthetic approaches for the preparation of **1**, **2** and **21** were recently reviewed [12].

**Scheme 1:**
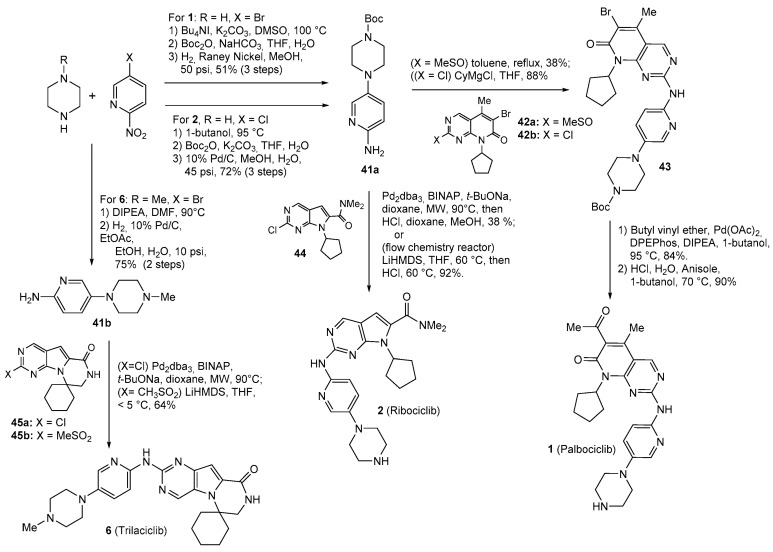
Synthesis of Palbociclib (**1**), Ribociclib (**2**) and Trilaciclib (**6**).

The preparations of **1** and **2** involve the same building block, *t*-butyl 4-(6-aminopyridin-3-yl)piperazine-1-carboxylate (**41a**), which was obtained through SN_Ar_ starting with piperazine and 2-nitro-5-halopyridine, followed by *N*-protection and catalytic hydrogenation (Scheme 1) [13,14]. In the discovery chemistry, the preparation of Palbociclib involved an SN_Ar_ reaction of **41a** with **42a** (X = MeSO) in toluene, but with an unsatisfactory yield (38%) [13]. Later, the synthetic procedure was optimized; the nucleophilicity of **41a** was improved by using a base (cyclohexyl magnesium chloride gave the best result) and by changing the leaving group of **42** from sulfoxide (**42a**) to chloride (**42b**, Scheme 1) [15]. The final compound **1** was obtained after Heck coupling on the bromine atom of **43** with butyl vinyl ether, followed by deprotection in an acidic medium [15,16].

Different from **1**, in the first synthesis of Ribociclib (**2**), compound **41a** was coupled with chloropyrimidine **44** through a palladium-catalyzed Buchwald–Hartwig amination reaction (Scheme 1) [17]. However, since the purification of the compound from the metal catalyst was troublesome, a transition metal-free synthesis was later developed and optimized for flow chemistry, involving the use of lithium bis(trimethylsilyl)amide (LiHMDS) as a base [18]. The synthesis of Trilaciclib (**6**) has evolved with similar chemistry. At first, **41b**, synthesized from *N*-methylpiperazine and 2-nitro-5-bromopyridine, was coupled with the chloro derivative **45a** using a Pd-catalyzed reaction [19], and after the optimization of the synthetic route, it was reacted with methylsulfone **45b** using LiHMDS as a base [20].

**Scheme 2:**
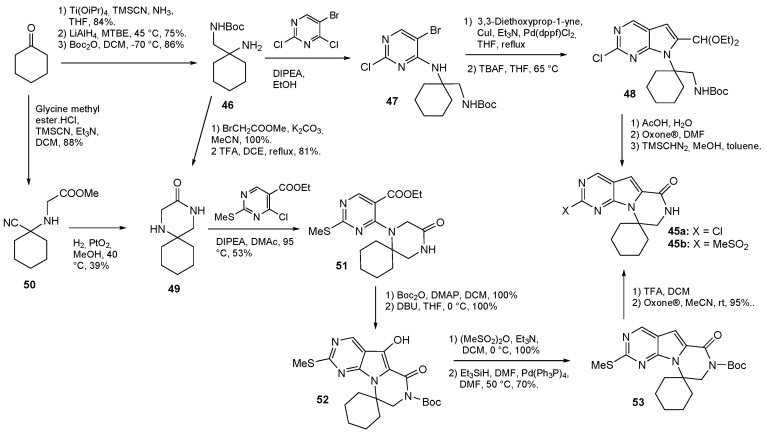
Synthesis of the 2-ketopiperazine derivative **45**.

Trilaciclib also contains a ketopiperazine moiety, whose synthesis is described in Scheme 2. In the first patent [19], the piperazinone ring of **45** was prepared via intramolecular condensation, forming the lactam moiety. In fact, diamine **46** (prepared in three steps from cyclohexanone) was condensed with 5-bromo-2,4-dichloropyrimidine, and on compound **47**, a Sonogashira reaction with 3,3-diethoxyprop-1-yne followed by ring closure afforded the protected aldehyde **48**. Elaboration of the aldehydic moiety to carboxylic ester, followed by *N*-Boc removal, afforded **45a**. In another patent [20], **45b** was prepared using a synthon, 1,4-diazaspiro[5.5]undecan-3-one **49**, in which the piperazinone ring was already formed. This compound was prepared in two different ways from cyclohexanone: (1) through the condensation of **46** with methyl bromoacetate, followed by *N*-deprotection leading to spontaneous amide bond formation; or (2) by reacting cyclohexanone with glycine methyl ester and trimethylsilylcyanide (TMSCN) to obtain **50**, which, after the nitrile reduction to amine, spontaneously formed the lactam ring, giving **49**. Condensation of **49** with ethyl 4-chloro-2-(methylthio)pyrimidine-5-carboxylate gave **51**; after the protection of the amidic NH with Boc anhydride, base-catalyzed intramolecular condensation afforded **52**. Removal of the phenolic group through the reduction of the triflate derivative gave **53**; *N*-deprotection and oxidation of the sulfide to sulfone finally led to **45b**.

The synthesis of **21** is reported in Section 2.2.

Vortioxetine (**3**) is an antidepressant agent with multiple activity on the serotoninergic system. The aim of Lundbeck’s researchers was the development of an antidepressant multitarget agent able not only to block the serotonin (5-HT) transporter (SERT), but also to activate the 5-HT1A receptor to obtain rapid autoreceptor desensitization, and to antagonize the 5-HT3 receptor to exert a positive effect on mood and cognitive impairment in patients with depression [21]. Later, it was found that **3** is also a partial agonist at the 5-HT1B subtype and an antagonist at the 5-HT7 and 5-HT1D receptors [22]. Vortioxetine increases neurotransmission in brain areas associated with major depressive disorder and also displays procognitive and antihyperalgesic activity [23,24].

**Scheme 3:**
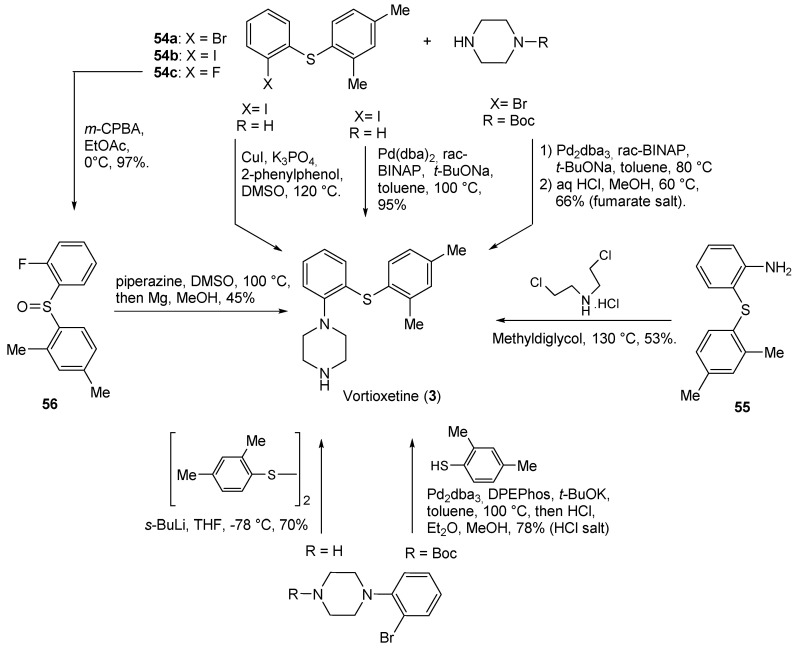
Synthesis of Vortioxetine (**3**).

Vortioxetine is a relatively simple molecule that can be prepared in many different ways. Some of the synthetic procedures that have been proposed to prepare **3** are reported in Scheme 3. The original method developed at Lundbeck involved the Buchwald–Hartwig addition of *N*-Boc-piperazine to (2-bromophenyl)(2,4-dimethylphenyl)sulfane **54a**, followed by acidic deprotection, obtaining **3** in a 66% yield [25]. Later, the same industry reported a different procedure, using commercially available 2-bromophenylpiperazine that involved the Pd-catalyzed addition of 2,4-dimethylphenylthiophenol on the bromine atom [21]. When the Buchwald–Hartwig reaction was performed between piperazine and the iodo derivative **54b**, the yield increased to 95%; unprotected piperazine and **54b** can also react in the presence of copper salts and a ligand (preferably 2-phenylphenol) [26]. Other methods that do not involve Pd catalysts have been described. The piperazine ring was built by reacting bis(dichloroethyl)amine with aniline **55** in a high-boiling solvent [27]; alternatively, bromo-lithium exchange on 2-bromophenylpiperazine and reaction with 1,2-bis(2,4-dimethylphenyl)disulfane afforded **3** in a 70% yield [28]. A method involving an SN_Ar_ reaction with piperazine is possible if an electron-withdrawing group is present on the ring (step g); after the oxidation of **54c** to **56** using *meta*-chloroperoxybenzoic acid (*m*-CPBA), the reaction of the sulfoxide **56** with piperazine and subsequent reduction with magnesium in methanol afforded **3** in a 45% yield [29].

Avapritinib (**4**) is a potent inhibitor of mutant forms of the protein tyrosine kinase KIT and of platelet-derived growth factor receptor alpha (PDGFRA), two membrane proteins belonging to the class III receptor tyrosine kinase subfamily. Avapritinib is indicated for the treatment of adults with metastatic gastrointestinal stromal tumors (GIST) harboring the PDGFRA D842V mutation; the drug is also used to treat systemic mastocytosis characterized by a KIT variant with the D816V mutation [30]. Such mutations are located in the activation loop and cause constitutive activity in the kinases, which become resistant to inhibitors binding to the inactive conformation, such as imatinib or sunitinib [31]. Avapritinib was developed by Blueprint Medicines, starting with the screening of a library of kinase inhibitors followed by optimization. Half-maximal inhibitory concentration (IC_50_) values of the activation loop mutants of KIT and PDGFRA reached the subnanomolar range, and the compound showed high selectivity for these proteins over several other kinases [32]. Avapritinib has also demonstrated inhibitory activity toward ABC drug transporters (ABCB1, P-glycoprotein, MDR1; ABCG2, BCRP) [33].

**Scheme 4:**
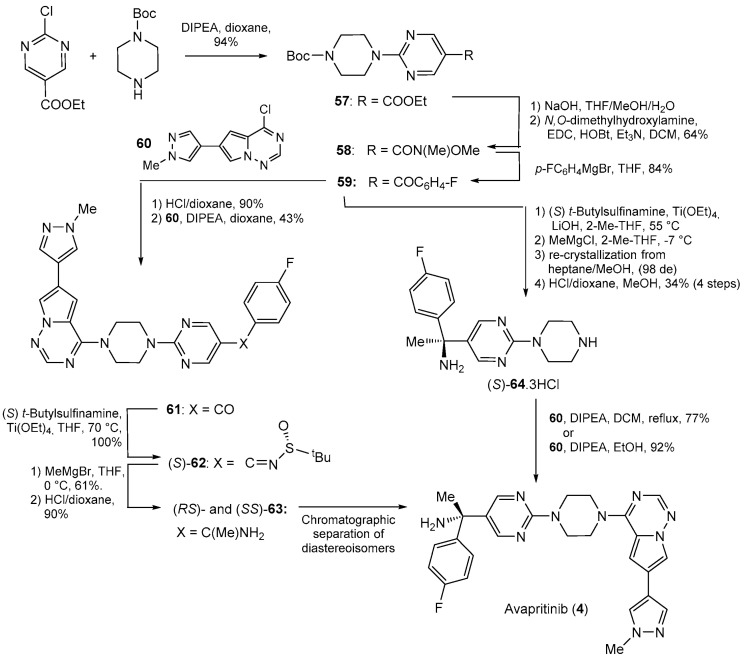
Synthesis of Avapritinib (**4**).

The synthesis of Avapritinib (Scheme 4) is reported only in patents. Both aryl groups on the piperazine N-atoms are aza-heterocycles, making SN_Ar_ reactions feasible. In fact, the initial intermediate **57** has been prepared in high yields by coupling *N*-Boc-piperazine and ethyl 2-chloropyrimidine-5-carboxylate. Transformation of the carbethoxy moiety of **57** into the Weinreb amide (**58**), followed by a Grignard reaction using 4-fluorophenyl magnesium bromide, gave the 4-fluorobenzoyl analog **59**, which was reacted, after acidic deprotection, with the chloro derivative **60** for the second SN_Ar_ reaction to obtain **61**. The addition of methyl magnesium bromide on the chiral sulfinamide (*S*)-**62**, followed by acidic hydrolysis and the chiral supercritical fluid chromatographic separation of the diasteroisomers, gave **4** [34]. The synthesis of **4** was later modified [35]. The procedure to insert the chiral center was performed on **59** and optimized to obtain (*S*)-**64** in high amounts without the need for expensive chromatographic separation. The yield of the reaction between (*S*)-**64** and **60** has been improved using ethanol in place of dichloromethane as a solvent [36].

Letermovir (**5**) is an antiviral agent against human cytomegalovirus (hCMV) infection. CMV is a herpes virus that causes severe morbidity and mortality in immunocompromised individuals, such as those with advanced human immunodeficiency virus (HIV) infection or those who have received solid organ or bone marrow transplants [37]. Letermovir was discovered by AiCuris through a high-throughput screening (HTS) campaign, aimed to find compounds with a mechanism of action that is different from the inhibition of DNA polymerase, which is the target of nucleoside–nucleotide analogs such as Ganciclovir or Cidofovir, commonly used to treat this infection [38]. Terminase is a fundamental protein complex in the DNA cleaving and packaging process; Letermovir is presumed to interfere with the interaction of viral concatemer DNA with the pUL56 subunit of the terminase complex [39]. Mutations at this level confer resistance to this drug, which in turn is active against viral strains resistant to CMV DNA polymerase inhibitors [37].

**Scheme 5:**
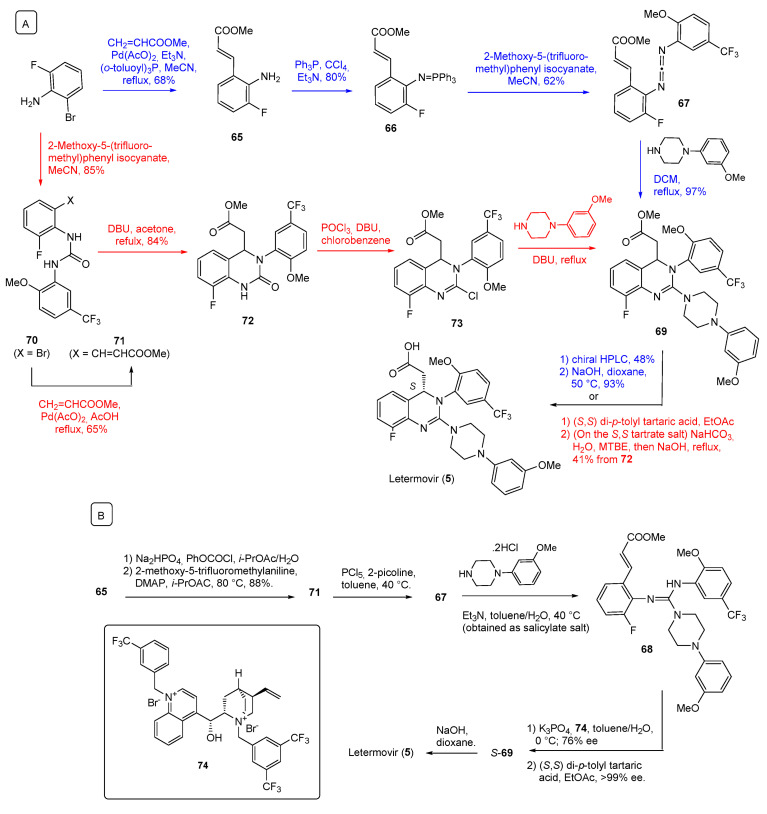
Synthesis of Letermovir (**5**) from 2-bromo-6-fluoroaniline ((**A**), discovery (blue) and first process (red) routes) and from compound **65** (**B**).

We included Letermovir in the diarylpiperazine group, although the quinazoline ring attached to one piperazine N-atom is partially hydrogenated. The reported synthetic procedures exploited the nucleophilic addition of commercially available 3-methoxyphenylpiperazine on a suitable acceptor, either carbodiimmide **67** (Scheme 5A, blue route) or dihydroquinazoline **73** (Scheme 5A, red route), both prepared starting with 2-bromo-6-fluoroaniline. The conditions used for the nucleophilic attack are crucial. In the discovery synthesis, carbodiimmide **67** was obtained through the Heck coupling of the starting aniline with methyl acrylate to give **65**, which was converted into the imminophosphorane **66** and then reacted with 2-methoxy-5-(trifluoromethyl)phenyl isocyanate [40]. The dihydroquinazoline **69** was obtained after the prolonged heating of **67** with (3-methoxyphenyl)piperazine, which behaved as a nucleophile and as a base to perform aza-Michael cyclization without isolating the guanidine intermediate **68** (structure shown in Scheme 5B). Ester hydrolysis and enantiomer separation by means of chiral high-performance liquid chromatography (HPLC) afforded **5**. Since this route was not suitable for scaling up, a new procedure was developed (Scheme 5A, red route) [41]. The starting aniline was reacted with 2-methoxy-5-(trifluoromethyl)phenyl isocyanate to give urea **70**, from which the tetrahydroquinazolinone **72** was obtained through Heck coupling with methyl acrylate under basic conditions without isolating the intermediate **71**. After the transformation of **72** into the chloro analog **73**, the arylpiperazine group was introduced via nucleophilic addition in the presence of 1,8-diazabicyclo[5.4.0]undec-7-ene (DBU) and under heating. Two crystallizations of the salt of racemic **69** with (*S*,*S*) di-(*p*-tolyl)tartaric acid, followed by basic hydrolysis, gave Letermovir (the *S* enantiomer) in about a 41% yield from **72**.

Later, a more efficient and cost-effective route (Scheme 5B) was developed for a long-term supply of the drug [42]. The transformation of **71** (synthesized from **65**) into **67** using phosphorous pentachloride and 2-picoline as a base, with careful control of the reaction conditions for the addition of (3-methoxyphenyl)piperazine, afforded **68** in a 90% yield, avoiding the formation of racemic ring-closure by-products. Aza-Michael cyclization was performed in the presence of the cinchona alkaloid analog **74** as a chiral phase transfer catalyst at a low temperature and by using potassium phosphate as a base. (*S*)-**69** was obtained with 76% ee, which was increased to >99% after crystallization of the (*S*,*S)* di-*p*-tolyltartrate salt. Basic hydrolysis afforded **5**.

Other compounds whose synthesis involves an SN_Ar_ reaction of *N*-alkylpiperazine on the suitable aromatic derivative are **7**–**11**. The aromatic substrate is a halobenzene carrying electron-withdrawing groups for **7** and **8** and an aza-heterocycle for **9**–**11**.

**Scheme 6:**
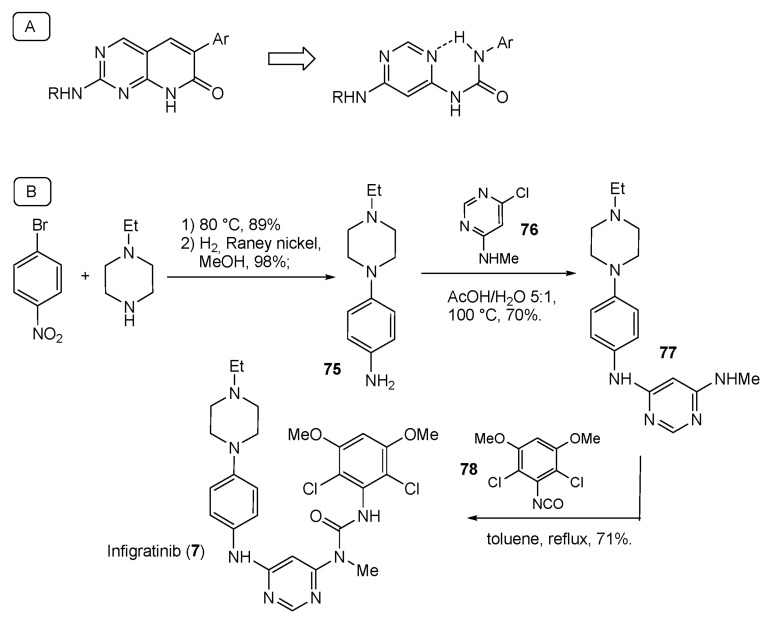
Design of 1-(6-(amino)pyrimidin-4-yl)-3-aryl-urea (**A**) and synthetic procedure to obtain Infigratinib (**7**) (**B**).

Infigratinib (**7**) is a fibroblast growth factor receptor (FGFR) inhibitor approved in 2021 for the treatment of cholangiocarcinoma, and it is under investigation for other malignancies characterized by abnormal activity of FGFRs [43]. The design of **7** by Novartis involved a ring-opening strategy to mimic the 6-aryl-pyrido[2,3-d]pyrimidin-7-one structure already found in promising protein kinase inhibitors. The pyridone ring was replaced with a urea moiety, and the substitution pattern on the pyrimidine ring was modified to obtain a structure mimicking the original one, owing to an intramolecular H-bond (Scheme 6A) [44]. Further optimization then led to Infigratinib, endowed with selectivity for FGFR 1–3 over several other kinases [45].

Compound **7** was synthesized through a four-step procedure starting with commercially available *N*-ethylpiperazine and 1-bromo-4-nitrobenzene. After condensation, followed by catalytic hydrogenation, intermediate **75** was treated with 6-chloro-*N*-methylpyrimidin-4-amine **76**. The urea linkage was formed through the reaction of **77** with the isocyanate **78**, obtaining **7** in a 71% yield (Scheme 6B) [45].

Entrectinib (**8**) is a kinase inhibitor approved for ROS1-positive metastatic non-small cell lung cancer (NSCLC) and neurotrophic receptor tyrosine kinase gene fusion positive solid tumors. Entrectinib, with similar potency, inhibits anaplastic lymphoma kinase (ALK), ROS1 and tropomyosin receptor kinase (TRK), showing antiproliferative activity in cancers originating from gene fusion mutations involving these proteins. Entrectinib is able to cross the blood–brain barrier, being effective against primary and metastatic brain tumors [46]. Entrectinib was discovered at Nerviano Medical Sciences, starting with the HTS of a corporate compound collection. The initial hit was optimized for the interaction with ALK; a kinase selectivity screening evidenced that the compound was also active on ROS1 and TRK [47].

**Scheme 7:**
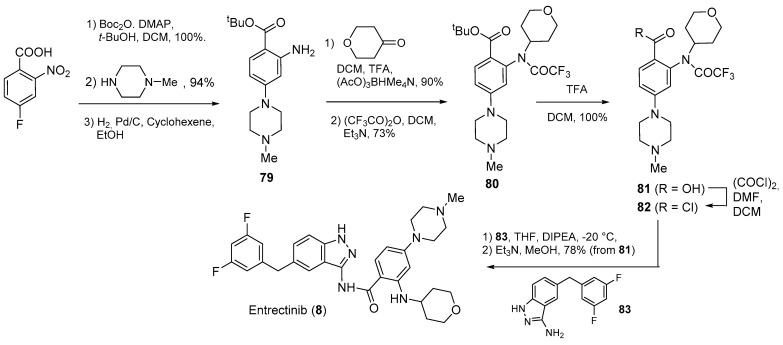
Synthesis of Entrectinib (**8**).

The synthesis of **8** (Scheme 7) started with commercially available 4-fluoro-2-nitrobenzoic acid, which was transformed into the *t*-butyl ester and then treated with *N*-methylpiperazine in excess, used as a reactant and solvent; reduction of the nitro group afforded **79**. Reductive amination using tetrahydropyran-4-one, followed by a reaction with trifluoroacetic anhydride, gave **80**, whose ester group was hydrolyzed under acidic conditions to **81** [48]. Treatment of **81** with oxalyl chloride gave the acyl chloride **82**, which was coupled with amine **83**; with this route, **8** was obtained in a 46% overall yield [47].

Avatrombopag (**9**) is a second-generation orally available thrombopoietin (TPO) receptor agonist approved by the FDA to treat thrombocytopenia in patients affected by chronic liver disease and scheduled to undergo an invasive procedure [49]. The design and development of **9** has not been published yet; there is evidence that **9** interacts with the TPO receptor in a binding site that is different from the endogenous agonist. In the transmembrane domain, a histidine residue at position 499 has been shown to be critical for Avatrombopag activity [50,51].

**Scheme 8:**
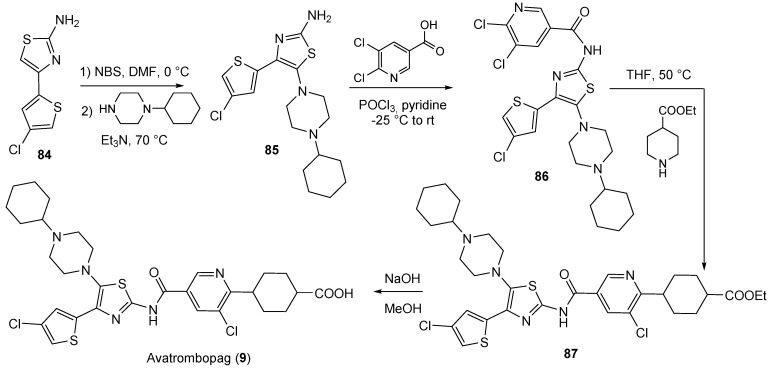
Synthesis of Avatrombopag (**9**). Reaction yields are not indicated in the original patent.

In Scheme 8, we report the synthesis of Avatrombopag as found in the original patent [52]. The synthesis started with 4-(4-chlorothiophen-2-yl)thiazol-2-amine **84**, which was transformed into the 5-bromo derivative and in situ reacted with *N*-cyclohexylpiperazine. The coupling of **85** with 5,6-dichloronicotinic acid gave **86**, which was treated with ethyl isonipecotate, obtaining **87**. Basic hydrolysis afforded **9**.

Netupitant (**10**) and Fosnetupitant (**11**) are neurokinin receptor 1 (NK1)-selective antagonists approved in combination with the 5-HT3 receptor blocker Palosetron to treat nausea and vomiting in patients undergoing cancer chemotherapy; the water-soluble **11**, the prodrug of **10**, is used as an intravenous (iv) formulation. The two-drug combination (called NEPA), administered in association with dexamethasone, proved to be effective both in the acute phase of chemotherapy-induced nausea and vomiting, mainly due to the activation of 5-HT3 receptors, and in the delayed phase, in which substance P stimulation of NK1 plays a major role [53].

**Scheme 9:**
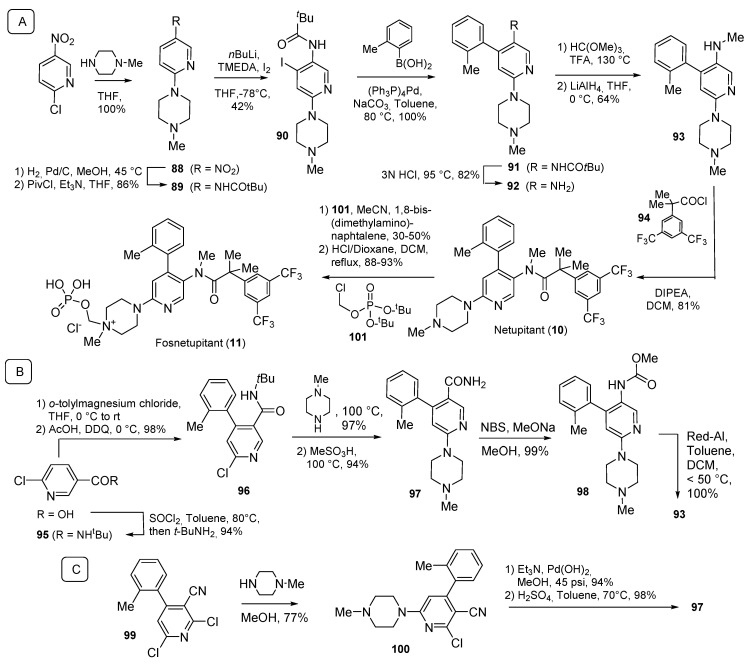
(**A**) Synthesis of Netupitant (**10**) and Fosnetupitant (**11**); Synthesis of compounds **93** (**B**) and **97** (**C**).

Three different synthetic procedures of Netupitant are reported in Scheme 9; commercially available *N*-methylpiperazine is always introduced through an SN_Ar_ reaction on a pyridine derivative. In the discovery synthesis (Scheme 9A), this reaction was performed on 2-chloro-5-nitropyridine with quantitative yields [54,55]. The nitro group of **88** was then transformed into pivaloylamide (**89**) in order to direct the following *ortho*-metalation-iodination sequence, leading to **90**. Subsequent Suzuki coupling gave **91**; acidic hydrolysis of the pivaloyl protective group led to amine **92**. Mono-methylation to afford **93** was achieved using a reductive *ortho*-ester procedure; then, the reaction with **94** gave **10** in a 15% overall yield. The drawbacks of this procedure were the lithiation reaction, for which a low temperature was necessary, the use of an expensive boronic acid reactant and a suboptimal yield.

The first process synthesis (Scheme 9B) started with commercially available 6-chloro-nicotinic acid, which was transformed into the *t*-butylamide **95**. The key step in this route was the 1,4-addition of a Grignard reagent, followed by oxidation of the dihydropyridine intermediate (not shown in Scheme 9) to obtain pyridine **96**. The addition of *N*-methylpiperazine was performed using the amine as a solvent; after the removal of the *t*-butyl group, **97** was obtained in a high yield [56]. The amide **97** was transformed into the carbamate **98** and reduced to **93**; the final acylation led to **10** in a 63% overall yield.

Another procedure was also developed (Scheme 9C) [57], based on a new, fast and productive synthesis of pyridine **99**; this new route can avoid the use of expensive 6-chloronicotinic acid and can bypass the Grignard addition step, whose work-up and purification are considered troublesome when applied to industrial quantities. The introduction of *N*-methylpiperazine into **99** was regioselective on the 6-Cl, giving **100** in a 77% yield. The 2-Cl was removed via hydrogenolysis using Pearlman’s catalyst, and the CN group was hydrolyzed to obtain amide **97**, which was transformed into **10**, as seen before.

Fosnetupitant **11** was prepared via alkylation of the *N*-methylpiperazine group with di-*t*-butyl (chloromethyl) phosphate **101**, followed by acidic hydrolysis of the *t*-butyl ester moiety; this method gave **11** with a yield of about 90% (Scheme 9A) [58].

Venetoclax (**12**) is a mimetic of the homology domain 3 (BH3) of B Cell Lymphoma protein (BCL), which antagonizes the activity of the pro-survival protein BCL-2, leading to the apoptosis of cancer cells. Venetoclax is approved to treat patients with chronic lymphocytic leukemia with a 17p deletion who received at least one prior therapy; it is also tested in clinical trials for other hematological malignancies [59]. The discovery of a BCL antagonist was addressed by Abbott by means of fragment-based drug discovery, but the development of **12** was troublesome due to difficulties improving BCL2 selectivity and optimizing the pharmacokinetic properties of the drug candidates [60,61].

**Scheme 10:**
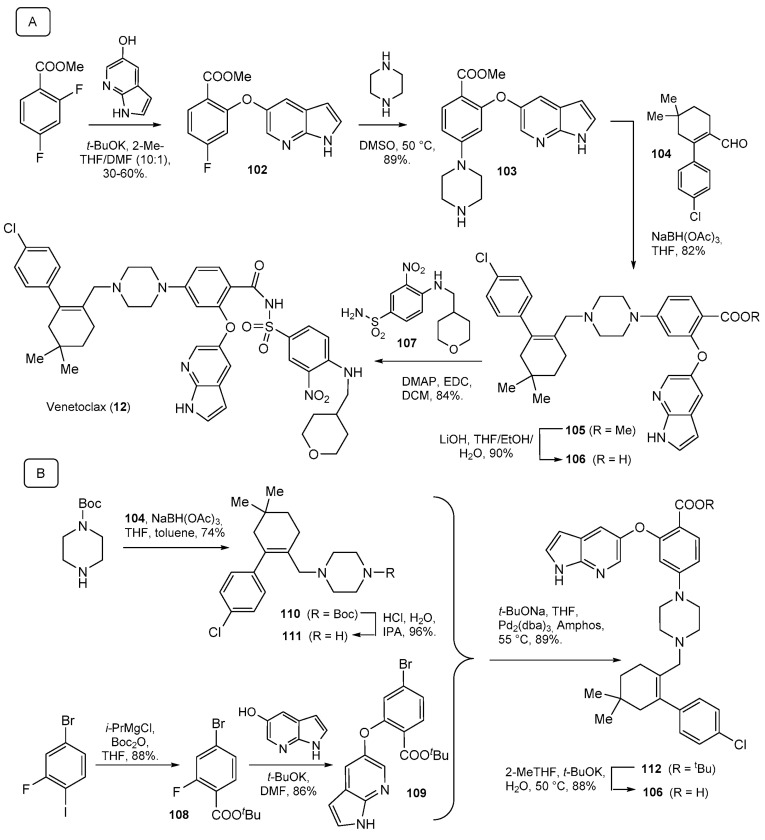
Synthetic procedures to obtain Venetoclax (**12**) (**A**) and compound **106** (**B**).

The first large-scale synthesis of Venetoclax was developed through the optimization of the reaction conditions used in the medicinal chemistry route (Scheme 10A) [62]. In the optimized procedure, unprotected piperazine was reacted with **102**, prepared with commercially available methyl 2,4-difluorobenzoate and 1*H*-pyrrolo[2,3-b]pyridin-5-ol. To minimize the formation of the double addition product, piperazine was used in excess (8 eq). Reductive amination of aldehyde **104** with **103** gave the ester **105**, which was hydrolyzed to **106** under basic conditions and coupled with sulfonamide **107**, obtaining **12**. Although the careful optimization of reaction conditions (time, solvent, temperature, equivalents of reactants) led to a synthetic route that was able to provide **12** on a multikilogram scale, it was not considered effective due to several drawbacks, such as a low overall yield and the formation of several impurities whose removal would increase the synthetic costs. In particular, concerns have been raised regarding the poor regioselectivity of the reaction of methyl 2,4-difluorobenzoate with hydroxyazaindole (first step in Scheme 10A). Therefore, the new route used *t*-butyl 2-fluoro-4-bromobenzoate **108**, conveniently prepared with 4-bromo-2-fluoro-1-iodobenzene, which gave a clean reaction with 1*H*-pyrrolo[2,3-b]pyridin-5-ol, leading to **109** (Scheme 10B). Another concern was the instability of aldehyde **104**, which complicated the purification of the intermediate compounds from impurities with mutagenic or carcinogenic potential. The main advancement in the new procedure was the involvement of a Buchwald–Hartwig amination reaction to obtain the *N*-arylpiperazine structure. Therefore, a freshly prepared solution of **104** was reacted with *N*-Boc-piperazine under a reductive amination protocol to give **110**, which was hydrolyzed to **111** and coupled with ester **109** using a Pd-catalyzed reaction. In this way, **112** was obtained with a high yield and purity. After hydrolysis to **106**, final coupling with **107** was performed as seen before, obtaining **12**.

Brexpiprazole (**13**) is a dopamine 2 receptor (D2) partial agonist approved by the FDA for the treatment of schizophrenia and as an adjunctive treatment for major depressive disorder. Brexpiprazole shows high affinity for several subtypes of serotonin, dopamine and noradrenaline receptors, behaving as a partial agonist on 5HT1A and antagonist on 5HT2A [63]. Brexpiprazole is a close analog of Aripiprazole (structure **127**), the first third-generation antipsychotic drug, from which it differs because of its lower intrinsic activity, which results in higher tolerability [64]. 

**Scheme 11:**
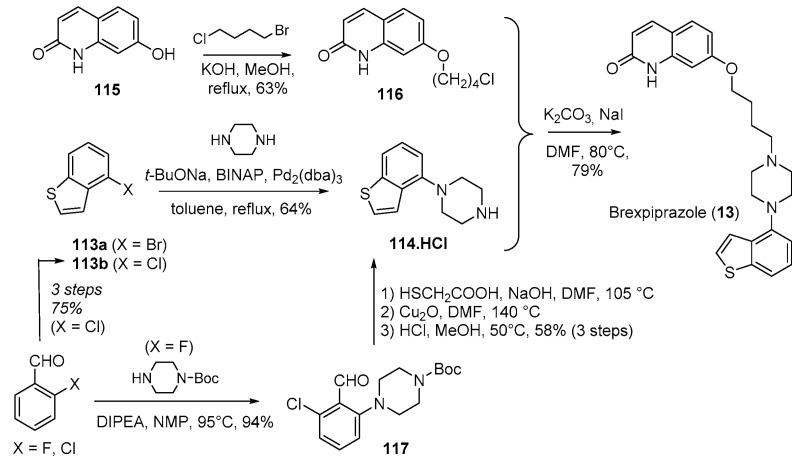
Synthesis of Brexpiprazole (**13**).

Also, the synthesis of **13** involved a Pd-catalyzed amination reaction. The first synthesis used by Otsuka Pharmaceuticals to prepare **13** looks very simple, since it consists of the separate preparation of intermediates **114** and **116** and their subsequent condensation (Scheme 11) [65]. The piperazine-containing intermediate, compound **114**, was prepared starting with **113a** and unsubstituted piperazine by means of a Pd-catalyzed amination reaction; **114** was obtained after chromatographic purification and was transformed into a hydrochloride salt. Compound **116** was prepared through the alkylation of **115** with 1-bromo-4-chlorobutane; the condensation of **114** with **116** using the Finkelstein reaction gave **13** in a 50% yield from **113a**. Besides the low yield, steps a and b produced a relatively large number of by-products whose removal was difficult. Otsuka Pharmaceuticals improved the synthetic method, developing routes to prepare **113b** starting with 2,6-dichlorobenzaldehyde [66] and trying different Pd catalysts for step a [67]. Kumar et al. reported that, although the yield of **114** was improved, the formation of by-products was not completely suppressed [68]. More recently, researchers from the Chinese Academy of Sciences developed, on a kilogram scale, a new method using 2-chloro-6-fluorobenzaldehyde. The nucleophilic displacement of fluorine with *N*-Boc-piperazine gave **117**; on this compound, the benzothiophene ring was assembled using thioglycolic acid, followed by decarboxylation and deprotection, obtaining **114**. Even if the total yield of **114** from 2-chloro-6-fluorobenzaldehyde was not high (54%), the impurities found in the formation of **117** were easily removed, yielding a high-purity compound [69].

Vilazodone (**14**) is a serotoninergic modulator approved for the treatment of major depressive disorder. This compound is endowed with serotonin reuptake inhibitory and 5-HT1A receptor agonist activities, with (sub)nanomolar IC_50_ values. This compound showed high selectivity for these targets with respect to other serotoninergic subtypes and to dopaminergic, adrenergic and histaminergic receptors [70].

**Scheme 12:**
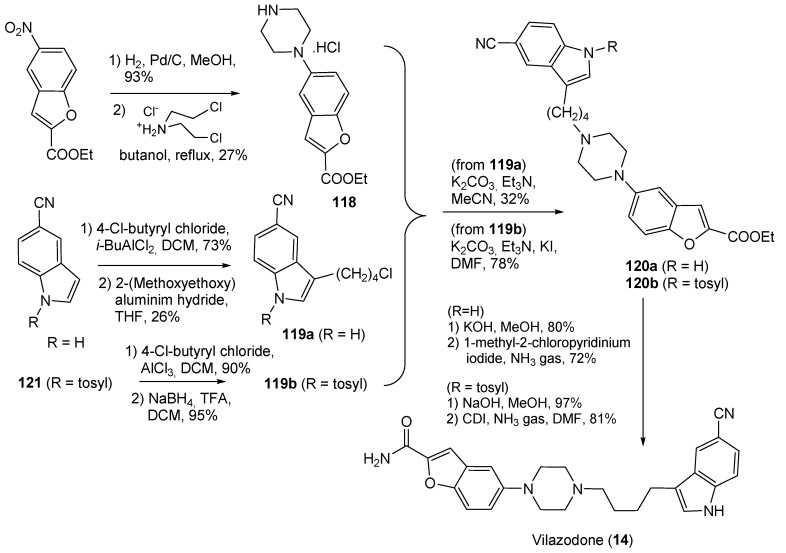
Synthesis of Vilazodone (**14**).

In the initial synthesis of Vilazodone (Scheme 12) [70], the piperazine ring was built using bis-(2-chloroethyl)amine and ethyl 5-aminobenzofuran-2-carboxylate, prepared through the reduction of the commercially available 5-nitro derivative. Compound **118** was then reacted with 3-(4-chlorobutyl)-1*H*-indole-5-carbonitrile **119a**, prepared with commercially available 5-cyanoindole; finally, the carbethoxy group of **120a** was transformed into carboxamide to obtain **14**. This procedure had several drawbacks due to the formation of various by-products and the use of expensive reagents, leading to a low yield, difficult purification and high costs. The procedure was then changed by protecting the indole NH with tosyl amide and by optimizing the reaction conditions due to the presence of the protecting group. In the reaction between **118** and **119b**, potassium iodide was added to improve the yield. The basic hydrolysis of the carbethoxy moiety, necessary for its transformation into carboxyamide, also removed the tosyl group [71].

Flibanserin (**15**) is a 5-HT1A receptor agonist approved for the treatment of low sexual desire disorder in premenopausal women. In vitro, this compound binds with high affinity to the 5-HT1A, dopamine D4 and 5-HT2A receptors, on which Flibanserin behaves, respectively, as an agonist, a very weak partial agonist and an antagonist [72]. A decrease in the serotonergic inhibition of excitatory neurotransmitters, dopamine and norepinephrine, is supposed to be the basis of its activity in female sexual desire. Flibanserin was originally developed as a treatment for depression [73].

**Scheme 13:**
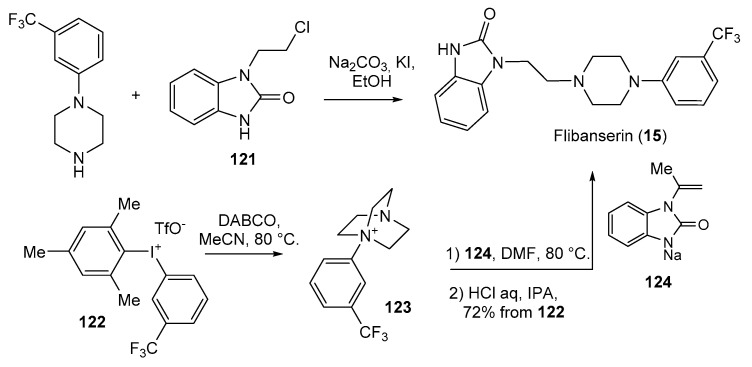
Synthesis of Flibanserin (**15**).

The original synthesis of **15** started with commercially available 1-[3-(trifluoromethyl)phenyl]piperazine, which was alkylated with 1-(2-chloroethyl)-1,3-dihydro-2*H*-benzo[d]imidazol-2-one **121** to give **15** (Scheme 13, yield not indicated in the patent) [74]. Subsequent efforts mainly regarded the optimization of the synthesis of **121** (see, for instance, ref. [75]). More recently, **15** was synthesized also by means of a new general method for the preparation of 1,4-disubstituted piperazines that exploits quaternary *N*-aryl-1,4-diazabyciclo[2.2.2]octane salts [76]. The addition of 1,4-diazabyciclo[2.2.2]octane (DABCO) to diaryliodonium triflate **122** gave the ammonium derivative **123**, which was reacted with benzimidazolone **124** (sodium salt) to obtain **15** after the acidic removal of the isopropenyl group.

Aripiprazole Lauroxyl (**16**) and Cariprazine (**17**) are antipsychotic drugs used in the treatment of schizophrenia. Compound **16** is the prodrug of Aripiprazole, a drug used since 2002 for the treatment of a wide variety of mood and psychotic disorders, and it has been developed into a long-acting injectable form. Compound **16** is metabolized into *N*-hydroxymethyl Aripiprazole and lauric acid; the former is then hydrolyzed into formaldehyde and Aripiprazole, achieving the maximal concentrations of the drug after about 41 days. This prodrug demonstrated improved medication adherence and reduced relapse rates [77,78]. Cariprazine **17** was obtained from the optimization of an impurity isolated during the large-scale synthesis of RG-15, an antipsychotic under development by Gedeon Richter and Forest Laboratories [79]. This compound is a D2/D3 receptor antagonist–partial agonist with preferential affinity for the D3 subtype; moreover, it also displays high affinity for the serotoninergic 5-HT2B receptor [80].

**Scheme 14:**
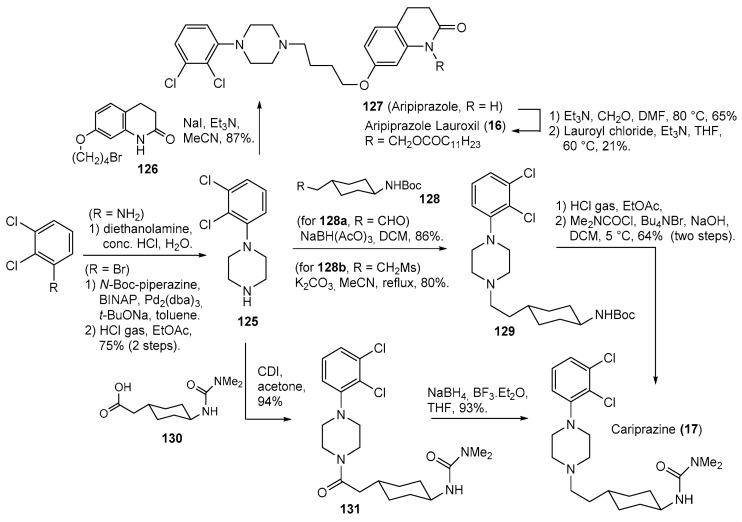
Synthesis of Aripiprazole Lauroxyl (**16**) and Cariprazine (**17**).

Both **16** and **17** were prepared starting with the same building block 1-(2,3-dichlorophenyl)piperazine **125**. In the original synthesis of Aripiprazole (Scheme 14) [81], **125** was prepared starting with 2,3-dichloroaniline and diethanolamine (according to ref [82], details are not given); reaction with the bromoalkyl derivative **126** gave Aripiprazole (**127**), which was converted into **16** via treatment first with formaldehyde and then with a lauric acid derivative (not indicated in the patent, presumably lauroyl chloride) [83].

For the synthesis of Cariprazine, Gedeon Richter and Forest Laboratories prepared **125** by means of a Buchwald–Hartwig reaction of *N*-Boc-piperazine on 1-bromo-2,3-dichlorobenzene, followed by acidic hydrolysis (Scheme 14) [84,85]. The *N*-Alkylaton of **125** was accomplished via reductive amination using aldehyde **128a** or via the alkylation of the mesylate **128b**, yielding **129**. After deprotection, the urea moiety was formed via reaction with dimethylcarbamic chloride [86,87]. Alternatively, amine **125** was acylated with acid **130** and 1,1′-carbonyldiimidazole (CDI), and the amide group of **131** was selectively reduced using sodium borohydride and boron trifluoride etherate to furnish **17 [88]**. The routes that use aldehyde **128a** or mesylate **128b** have similar yields (about 35%), and that involving acid **130** seems more efficient (57% yield). A recent paper reviewed all the synthetic methods used to prepare **17**, which mainly differ in their preparation of the cyclohexyl building blocks (**128** or **130**) [85].

### 2.2. N-Alkyl Derivatives

There are three important methods to transform piperazine into its *N*-alkyl analogs: nucleophilic substitution on alkyl halides or sulfonates, reductive amination [89] and the reduction of carboxyamides [90]. *N*-alkylpiperazines **12**–**23** have been prepared using these procedures. As we have just seen, all three of these methods have been used for the preparation of **17** using different synthetic routes developed by the originator company (Gedeon Richter and Forest Laboratories) (Scheme 14).

**Scheme 15:**
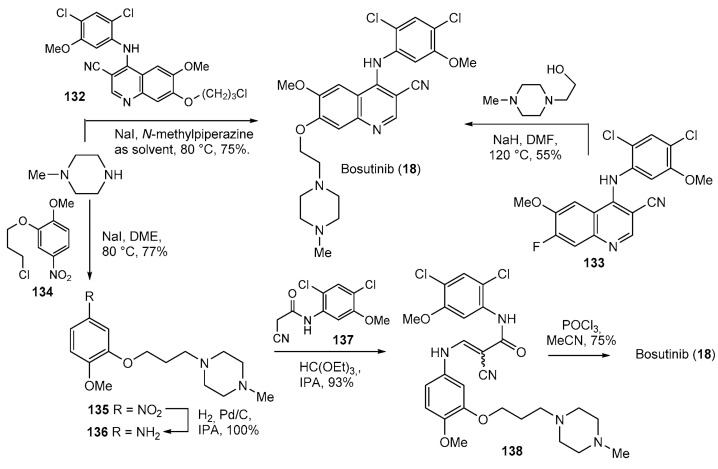
Synthesis of Bosutinib (**18**).

*N*-alkylation using alkyl chlorides or bromides has been used for the synthesis of compounds **13**–**16** and **18**, shown in Scheme 11, Scheme 12, Scheme 13, Scheme 14 and Scheme 15. In these cases, the addition of sodium or potassium iodide, which promotes halogen exchange and increases the reactivity of the leaving group, was performed in order to improve the yield (see, for instance, the synthesis of **14**, Scheme 12) or to avoid reaction conditions that are too harsh.

*N*,*N′*-dialkylpiperazine **18**–**23** are kinase inhibitors used in the treatment of various types of cancers. Bosutinib (**18**) is a dual ABL/SRC inhibitor, and Ponatinib (**19**) is a BCR-ABL inhibitor. Both drugs are used for Imatinib-resistant chronic myelogenous leukemia (CML); different from **18**, **19** is able to inhibit the T315I mutant enzyme [91]. Nintedanib (**20**) was approved for the treatment of NSCLC and for idiopathic pulmonary fibrosis. Nintedanib inhibits different angiokinases (PDGFR, FGFR and vascular endothelial growth factor (VEGFR)) but also non-receptor kinases [92].

In the synthetic routes originally developed at Wyeth for Bosutinib (**18**), the piperazine ring was inserted in the last synthetic steps, in two different ways (Scheme 15): through the nucleophilic attack of *N*-methylpiperazine on the 3-chloropropyl derivative **132** using the amine as a solvent, or through the addition of commercially available 1-(3-hydroxypropyl)-4-methylpiperazine on the aryl fluoride **133** using sodium hydride to increase the nucleophilicity of the OH group [93,94]. The procedure used for the manufacture of late-stage clinical supplies, on the contrary, inserted the piperazine moiety as the first step via the reaction of *N*-methylpiperazine with the chloroalkyl derivative **134**, obtaining **135**, which was reduced to **136**. The quinoline ring of **18** was built on the amino group via reaction with cyanoacetamide **137** and triethyl orthoformate, obtaining **138** as a mixture of cis/trans isomers; cyclization using phosphorus oxychloride in acetonitrile provided **18 [95]**. In both routes, the reaction of *N*-methylpiperazine was facilitated by the addition of sodium iodide.

**Scheme 16:**
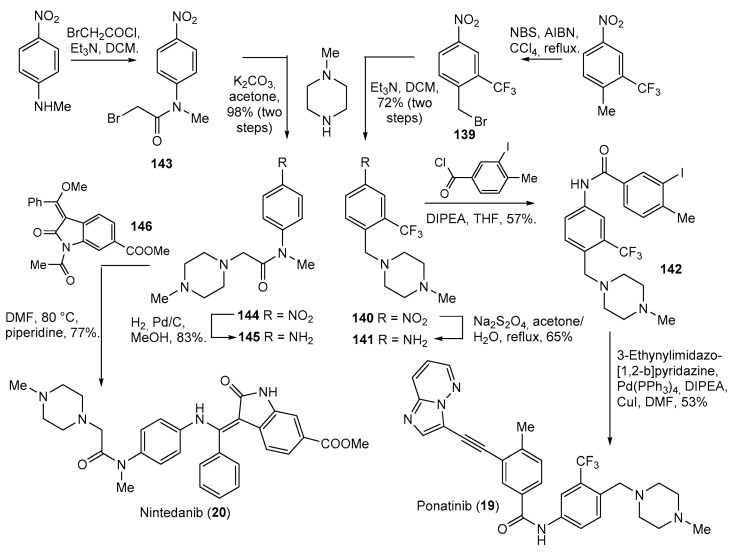
Synthesis of Ponatinib (**19**) and Nintedanib (**20**).

The syntheses of Ponatinib (**19**) and Nintedanib (**20**) required piperazine *N*-alkylation using reactive alkyl halides; therefore, there was no need to add an iodide salt. The original procedures involved the addition of commercially available *N*-methylpiperazine on benzyl bromide **139** or on bromoacetyl derivative **143** (Scheme 16). For the synthesis of **19**, benzyl bromide **139** was prepared via the bromination of commercially available 1-methyl-4-nitro-2-(trifluoromethyl)benzene followed by the reaction with *N*-methyl-piperazine, obtaining **140**. Reduction of the nitro group and the reaction of amine **141** with 3-iodo-4-methylbenzoyl chloride gave **142**, which was coupled with 3-ethynylimidazo[1,2-*b*]pyridazine under Sonogashira conditions, obtaining **19** [96]. Compound **143** was prepared through the acylation of *N*-methyl-4-nitroaniline with bromoacetyl bromide. After the addition of *N*-methylpiperazine, the nitro group of **144** was hydrogenated, and aniline **145** was coupled with **146** to yield **20** [97,98].

Also, the synthesis of Maralixibat (**24**) involves *N*-alkylation using a reactive alkyl halide; in this compound, the piperazine ring is part of a DABCO moiety, with one N-atom being quaternary. Maralixibat is an ileal bile acid transporter (IBAT) inhibitor approved for the treatment of rare cholestatic liver diseases, including Alagille syndrome, progressive familial intrahepatic cholestasis and biliary atresia. Maralixibat is a quaternary ammonium compound that is minimally absorbed and interacts with the transporter in the ileal lumen. IBAT inhibition reduces bile acid reabsorption and increases their elimination with feces. As a consequence, the serum levels of bile acids are reduced, and so is the risk of bile acid-mediated liver damage [99].

**Scheme 17:**
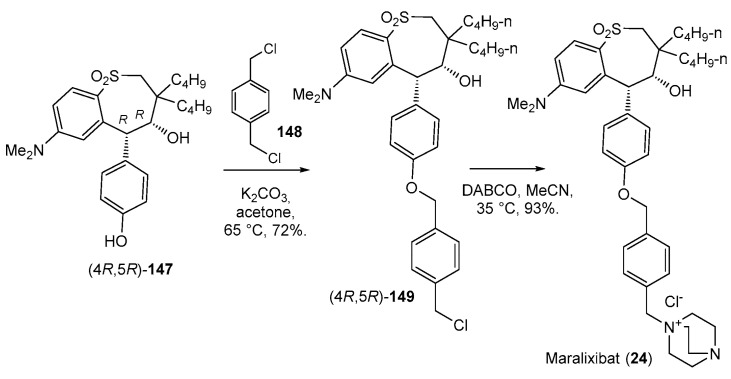
Synthesis of Maralixibat (**24**).

The most laborious part of the synthesis of **24** is related to the preparation of the chiral benzothiazepine oxide (4*R*,5*R*)-**147** (not addressed), and the insertion of the piperazine moiety simply involved the nucleophilic addition of DABCO to the reactive benzylic chloride **149** (Scheme 17), prepared via the alkylation of compound **147** with 1,4-α,α′-dichloro-*p*-xylene **148**. The quaternary ammonium nature of **24** facilitated its purification since this compound readily precipitated from the reaction mixture [100,101].

**Scheme 18:**
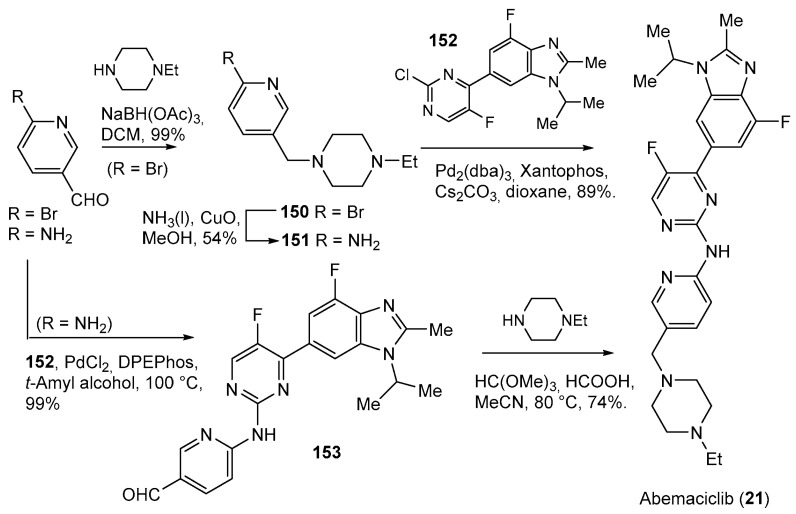
Synthesis of Abemaciclib (**21**).

**Scheme 19:**
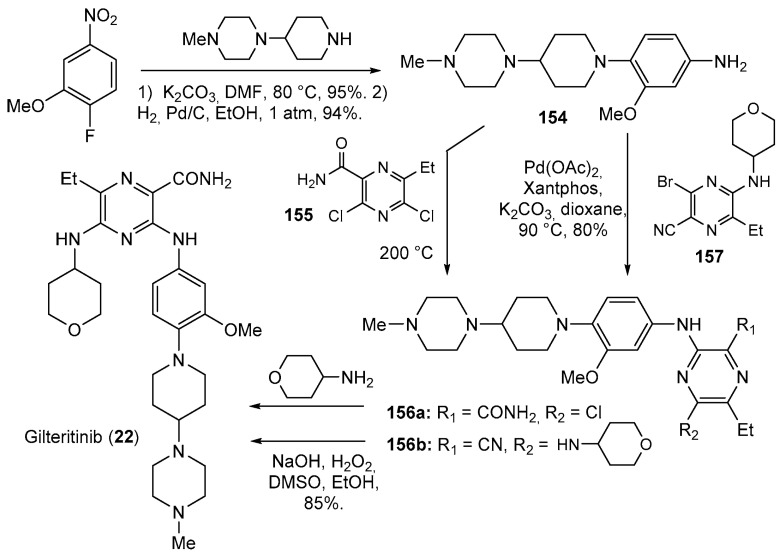
Synthesis of Gilteritinib (**22**).

Reductive amination has been used to prepare the *N*-alkyl compounds **12**, **16**, **21** and **22** using the suitable aldehyde and sodium triacethoxyborohydride. The syntheses of **12** and **16** are reported in Scheme 10 and Scheme 14, respectively, and those of **21** and **22** are shown in Scheme 18 and Scheme 19, respectively.

The CDK 4/6 inhibitor Abemaciclib (**21**) is described in Section 2.1. Its synthesis (Scheme 18) started with commercially available 6-bromonicotinaldehyde. Reductive amination using sodium triacethoxyborohydride and *N*-ethylpiperazine gave **150**, which was transformed into aniline **151** and reacted with 2-chloropyrimidine **152**, yielding **21** [102]. Alternatively, compound **152** was condensed with 6-aminonicotinaldehyde using Pd-catalyzed coupling, and then **153** was treated with *N*-ethylpiperazine using Leuckart–Wallach conditions [103,104]. In this case, the use of triacethoxyborohydride was discarded since it produced a small percentage of a by-product, the alcohol deriving from the reduction of aldehyde **153**, which was difficult to eliminate during purification.

**Scheme 20:**
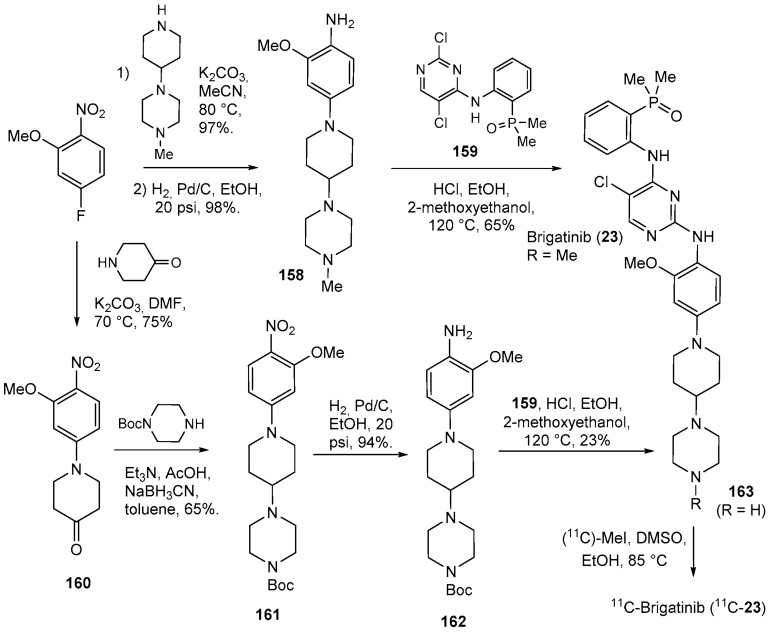
Synthesis of Brigatinib (**23**) and its ^11^C-analog.

Gilteritinib (**22**) is indicated for the treatment of acute myeloid leukemia deriving from an FMS-like tyrosine kinase gene (FLT3) mutation detected by a companion diagnostic; it is a dual FLT3/AXL selective inhibitor [105]. Brigatinib (**23**) is an ALK inhibitor, carrying an unusual dimethylphosphine oxide moiety as an H-bond acceptor [106]; it is approved to treat ALK-positive metastatic NSCLC characterized by the presence of an EMAP Like 4 (EML4)−ALK fusion protein. Both Gilteritinib and Brigatinib carry the 1-methyl-4-(piperidin-4-yl)piperazine group; their original synthetic routes used this commercially available reagent (Scheme 19 and Scheme 20).

The synthesis of **22** was reported by Astellas only in patents, from which it is difficult to extract the relevant information on reaction conditions and yields [107]. The description of the original route was obtained from another source [108], which reports that the reaction of compound **154** with pyrazine **155** at high temperatures (150–200 °C), followed by the treatment of **156a** with tetrahydro-2*H*-pyran-4-amine, gave **22** in only a 25% yield (Scheme 19). Differently, the method reported in patent CN106083821A [108] involved the Pd-catalyzed amination of **157** with **154**, obtaining **156b**; the hydrolysis of the nitrile group to carboxyamide gave **22**. The total yield was higher with the new route. The preparation of **154** (reaction of 1-methyl-4-(piperidin-4-yl)piperazine with 1-fluoro-2-methoxy-4-nitrobenzene followed by catalytic hydrogenation) was reported by Astellas in ref. [109]

For the synthesis of **23** (Scheme 20), similar chemistry was applied to obtain **158**, which was reacted with 2-chloropyrimidine **159** to obtain the final compound [106]. Very recently, the ^11^C analog of **23** was described as a positron emission tomography (PET) radiotracer to assess the mutational status of Brigatinib’s target kinases and to predict the benefit from the treatment of NSCLC patients [110]. A free NH group on piperazine was required before making the final radiolabeling step; therefore, the 4-piperidinyl-piperazine moiety was built by reacting 4-fluoro-2-methoxy-1-nitrobenzene with 4-piperidone, obtaining **160**, and by performing reductive amination using *N*-Boc-piperazine and sodium cyanoborohydride in the presence of acetic acid. The nitro group of **161** was hydrogenated, and **162** was coupled with **159** in the usual way. The alkylation of **163** with (^11^C)-methyl iodide gave the desired compound (10% radiochemical yield).

The preparation of the last two *N*-alkylpiperazine derivatives (Mitapivat **25** and Zavegepant **26**) is described in Section 2.3.

### 2.3. N-Acyl Derivatives

Compounds **25**–**28** were prepared by reacting the appropriate piperazine derivative with a suitably activated carboxylic acid. These procedures were straightforward but required optimization specially for large-scale processes.

**Scheme 21:**
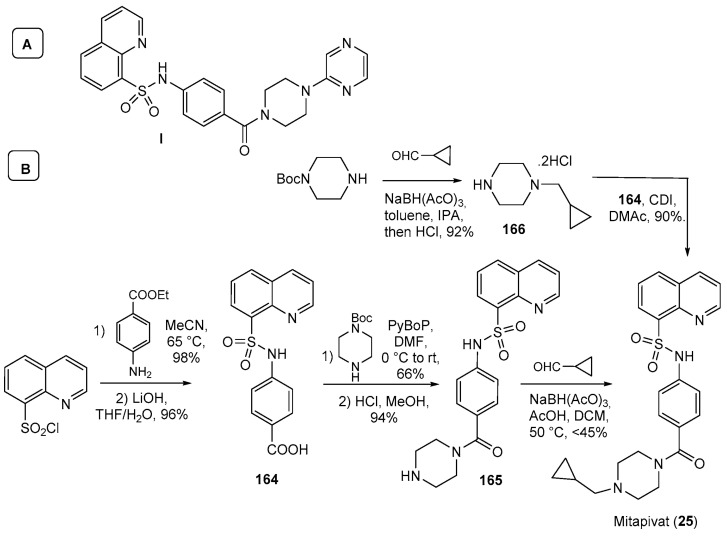
Structure of the PKM2-activator **I** (**A**) and synthesis of Mitapivat (**25**) (**B**).

Mitapivat (**25**) is a pyruvate kinase activator approved to treat hemolytic anemia in pyruvate kinase (PK) deficiency. PK catalyzes the final step in glycolysis, converting phosphoenolpyruvate to pyruvate, producing adenosine triphosphate (ATP). Mitapivat was obtained starting with quinoline-8-sulfonamides I (Scheme 21A), an activator of the PK-M2 isoform found in muscle cells [111]. The pyrazine moiety was replaced with a cyclopropylmethyl group in order to obtain activating properties on the isoform expressed in the red blood cell (PK-R). Mitapivat binds to an allosteric site, distinct from the pocket occupied by fructose bisphosphate (the natural enzyme activator), and it is able to trigger both the wild type and the mutated isoforms [112,113].

The synthesis of Mitapivat is shown in Scheme 21B. Quinoline-8-sulfonyl chloride was reacted with ethyl 4-aminobenzoate, and the ester function was hydrolyzed to give **164**. Amide **165** was prepared by coupling **164** with *N*-Boc-piperazine using (benzotriazol-1-yloxy)tripyrrolidinophosphonium hexafluorophosphate (PyBoP) as a coupling reagent, followed by acidic deprotection. The reaction of **165** with cyclopropanecarbaldehyde and sodium triacetoxyborohydride gave the final compound with a yield below 45% [114]. In a following patent, Agios Pharmaceuticals improved the process by reacting **164** with CDI and 1-(cyclopropylmethyl)piperazine **166** (prepared by means of reductive amination with *N*-Boc-piperazine and cyclopropanecarbaldehyde) [115].

Zavegepant (**26**) is a calcitonin gene-related peptide (CGRP) receptor antagonist with high affinity for its receptor (Ki 23 pM) and high potency in reverting the CGRP-induced dilation of ex vivo human intracranial arteries (EC_50_ 880 pM). Zavegepant is administered as a nasal spray formulation, owing to its good water solubility, and it is approved for the acute and/or preventive treatment of migraines [116].

**Scheme 22:**
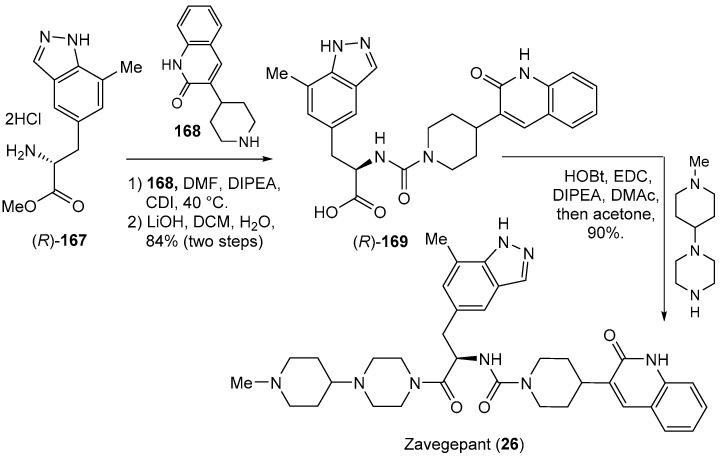
Synthesis of Zavegepant (**26**).

The final steps of the optimized synthesis of **26** are shown in Scheme 22. Considerable efforts have been spent on the synthesis of the chiral aminoacid *R*-**167** [117] and of the piperidine **168** [118], which were coupled using CDI to afford urea **169** after the hydrolysis of the carbomethoxy group. The insertion of the piperazine moiety was the last step, using commercially available 1-(1-methylpiperidin-4-yl)piperazine, 1-ethyl-3-(3-dimethylaminopropyl)carbodiimide (EDC) and 1-hydroxybenzotriazole (HOBt) as coupling reagents. Whereas the synthesis of urea *R*-**169** required careful optimization, due to the ambident nucleophilicity of **167**, the last step was more straightforward. To maximize process throughput, the final product (as HCl salt) was precipitated directly from the reaction mixture in *N*,*N*-dimethylacetamide (DMAc) by adding acetone [117].

Olaparib and Fostemsavir are diacylpiperazine derivatives. Olaparib (**27**) is a poly-ADP-ribose-polymerase (PARP) inhibitor approved for the treatment of Breast-Related Cancer Antigens (BRCA)-associated tumors, such as ovarian, breast, pancreatic and prostate cancer. PARP inhibition induces synthetic lethal interactions in cancer cells already deficient in DNA repair mechanisms, resulting in cell death. Olaparib interacts with the nicotinamide-binding pocket of the enzyme; the phtalazinone ring mimics the amide group of nicotinamide [119]. The main role of the cyclopropyl(piperazin-1-yl)methanone moiety is to increase water solubility and oral bioavailability [120], but the carbonyl group is also engaged in a water-mediated H-bond with the target.

**Scheme 23:**
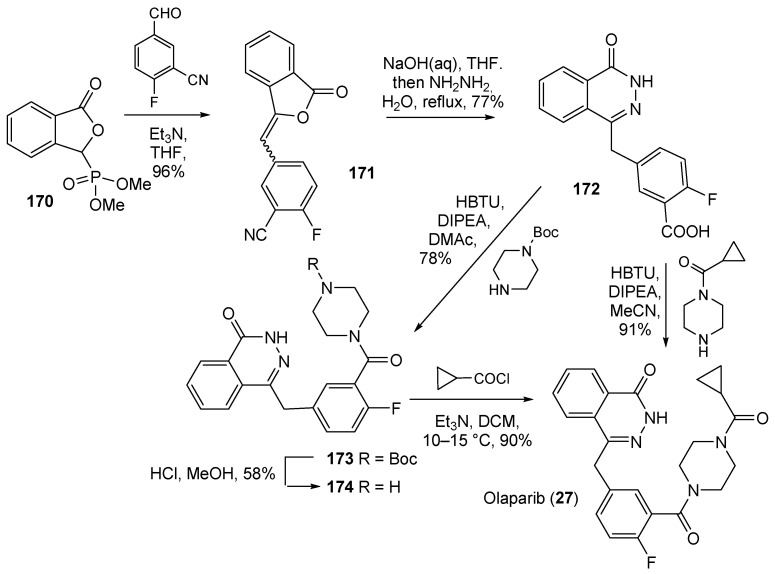
Synthesis of Olaparib (**27**).

For the synthesis of Olaparib, acid **172** was prepared starting with 3-dimethoxyphosphoryl-3*H*-2-benzofuran-1-one **170** through condensation with 2-fluoro-5-formylbenzonitrile, followed by the hydrolysis of the nitrile group of **171** and treatment with hydrazine hydrate (Scheme 23). The piperazine moiety was attached by reacting **172** with cyclopropyl(piperazin-1-yl)methanone in acetonitrile or by using *N*-Boc-piperazine with the same coupling reagents but in a different solvent (DMAc), followed by the deprotection of **173** and the reaction of **174** with cyclopropanecarbonyl chloride [121,122]. This second route was also applied to the synthesis of Olaparib derivatives carrying groups that were different from the cyclopropanecarbonyl one, which could be potentially useful as imaging agents [123].

**Scheme 24:**
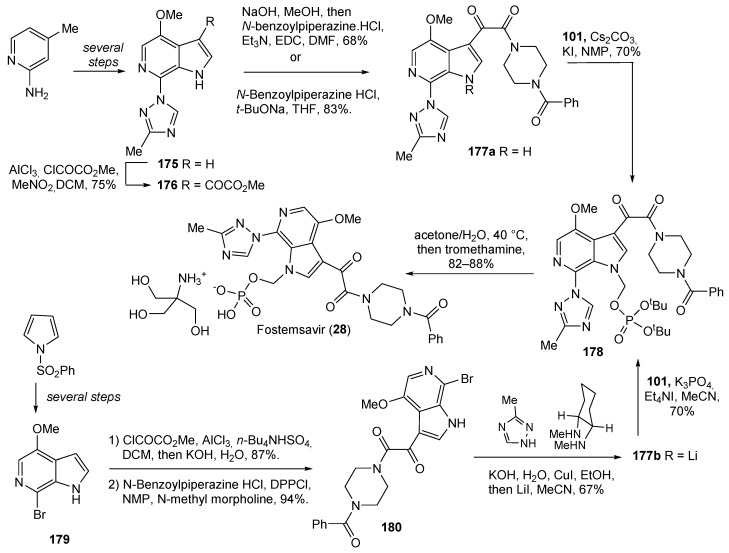
Synthesis of Fostemsavir (**28**).

Fostemsavir (**28**) is the prodrug of Temsavir (**177a**, structure shown in Scheme 24), an HIV attachment inhibitor, which blocks the interaction of the surface envelope protein gp120 with the CD4 receptor, thus preventing virus entry into the cells; this mechanism of action is different from that of other virus entry inhibitors such as Enfuvurtide and Maraviroc [124]. Fostemsavir is endowed with high aqueous solubility; its administration increases the plasma levels of Temsavir with respect to the administration of the parent molecule. The prodrug is hydrolyzed by an alkaline phosphatase in the gut, releasing the parent drug before absorption, resulting in very low systemic exposure of **28** [125].

The synthesis of Fostemsavir is shown in Scheme 24. Several routes are described, which require careful optimization for scaling up, owing to the complex reactivity of the azaindole moiety (see ref. [125] and the references cited therein). Here, only the procedure used in the medicinal chemistry and the commercial one are discussed. In the discovery chemistry [126], **28** was obtained starting with 2-aminopicoline, which was transformed into **175**; Friedel–Crafts acylation was performed on this compound using methyl 2-chloro-2-oxoacetate. The hydrolysis of ester **176** and reaction with commercially available *N*-benzoylpiperazine using EDC as a coupling reagent gave amide **177a** (Temsavir). Alkylation with di-*t*-butyl (chloromethyl) phosphate **101** and the hydrolysis of the ester groups of **178** gave **28**, which was precipitated as tromethamine salt via the addition of acetone.

Differently, the optimized industrial synthesis started with 1-(phenylsulfonyl)-pyrrole, which was converted into the bromo-derivative **179**, suitable for the acylation reaction necessary to insert the oxalyl moiety. The safety concerns connected to the large-scale use of nitromethane (as in step a, Scheme 24) were solved by using tetra-*n*-butylammonium hydrogen sulfate to increase solvent polarity and favor AlCl_3_ dissolution. After the hydrolysis of the ester group, amidation was performed using diphenylphosphinic chloride (DPPCl) as a coupling reagent, obtaining **180**. Regioselective copper-mediated Ullmann–Goldberg–Buchwald coupling introduced the triazole group on **180**, and the addition of lithium iodide allowed obtaining **177b**, which, different from **177a**, was crystalline and easier to purify. Tetraethylammonium iodide was added in the reaction with **101**, owing to the low reactivity of **177b** in this step. In the majority of the synthetic routes developed at Bristol Myers Squibb, the attachment of *N*-benzoylpiperazine was performed starting with the acid and using a suitable coupling reagent. In one route, **177a** was prepared by reacting ester **176** directly with the sodium salt of *N*-benzoylpiperazine to increase nucleophilicity (Scheme 24) [127]. Even though the yield reported for this step was high, the method involving ester hydrolysis was preferred in the optimized route.

## 3. Synthesis of Drugs Carrying *C*-Substituents on the Piperazine Ring

Commercially available piperazine reagents with *C*-substituents have been used to prepare compounds **29**–**31**; therefore, the synthetic routes involve the same methods discussed previously for *N*-alkyl and *N*-aryl derivatives.

Selpercatinib (**29**) is a rearranged during transfection (RET) receptor tyrosine kinase inhibitor approved for cancers harboring RET mutations (metastatic RET fusion-positive NSCLC, advanced/metastatic RET-altered medullary thyroid cancer and papillary thyroid carcinoma). Selpercatinib binds to the ATP site; its unique mode of binding allows the interaction with the RET protein to avoid steric clashes with gatekeeper mutations at V804. However, mutations in other parts of the protein confer resistance to this drug [128,129].

**Scheme 25:**
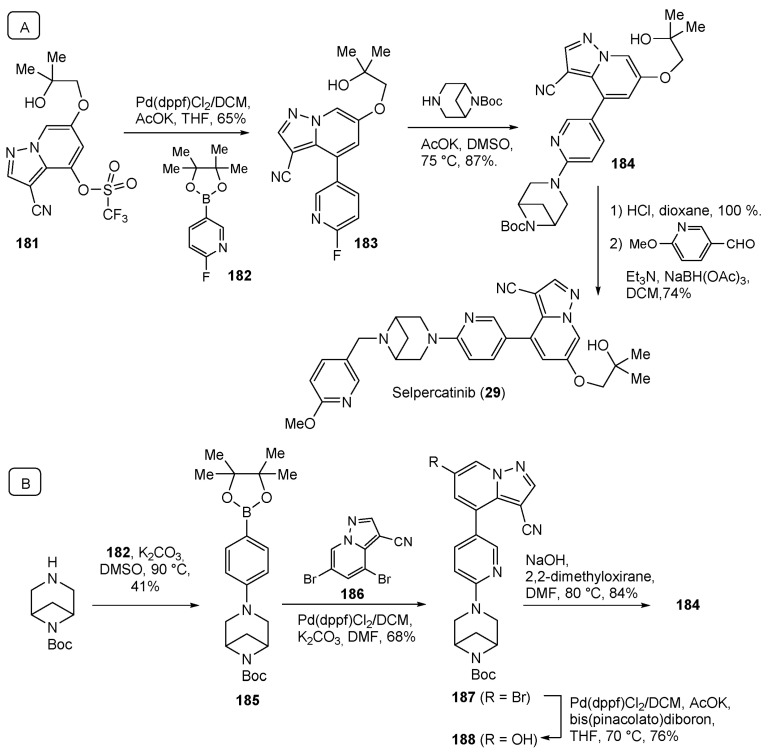
Synthetic procedures to obtain Selpercatinib (**29**) starting from **181** (**A**) or from **182** (**B**).

In **29**, the piperazine ring is included in a diazabicycloheptane group; the two different synthetic routes developed by Array BioPharma exploited an SN_Ar_ reaction to insert this moiety. Suzuki coupling between pyrazolo[1,5-a]pyridin-4-yl trifluoromethanesulfonate **181** and 2-fluoro-5-(4,4,5,5-tetramethyl-1,3,2-dioxaborolan-2-yl)pyridine **182** gave **183**, which was reacted with commercially available *N*-Boc-3,6-diazabicyclo[3.1.1]heptane, obtaining **184** (Scheme 25A). Removal of the protecting group and reductive amination using 6-methoxynicotinaldehyde gave **29**. Alternatively, **184** was prepared starting with the dibromo derivative **186** (Scheme 25B). Borolane **185**, obtained by condensing *N*-Boc-3,6-diazabicyclo[3.1.1]heptane with fluoropyridine **182**, was reacted with **186**, and another Suzuki reaction was performed to replace the 6-bromine of **187** with OH. The alkylation of phenol **188** with 2,2-dimethyloxirane gave **184**. The two methods afforded **29** with a similar yield (about 41% starting with **181** or **186**) [130,131].

Risdiplam (**30**) is an orally active splicing modifier, approved for the treatment of spinal muscular atrophy. It promotes the inclusion of the survival of motor neuron 2 (SMN2) exon 7 in the messenger RNA to produce a functional SMN protein in individuals lacking the SMN1 isoform. In **30**, the 4,7-diazaspiro[2.5]octane moiety has the dual role of optimizing physicochemical properties (basicity, lipophilicity) and contributing to binding with the target molecules through the interaction of the protonated NH atom [132,133].

**Scheme 26:**
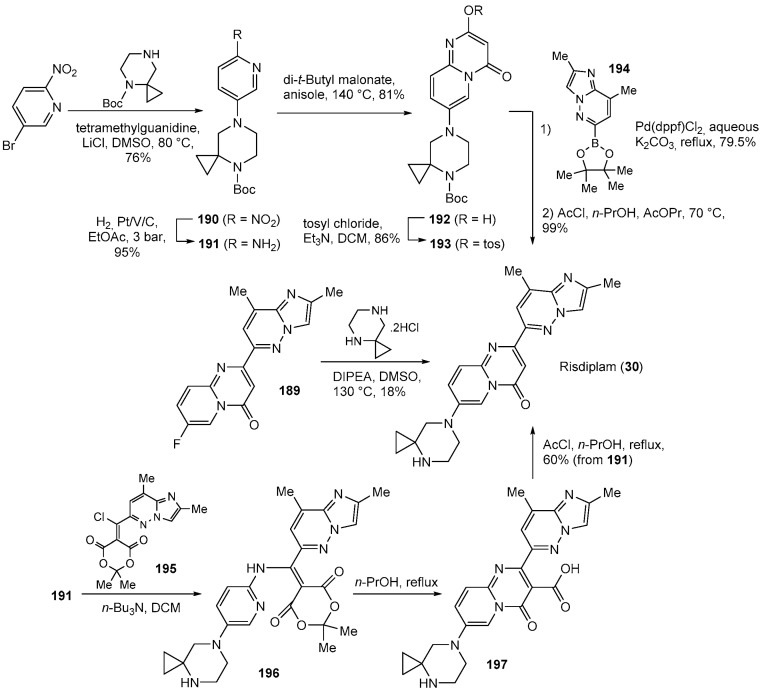
Synthesis of Risdiplam (**30**).

In the medicinal chemistry synthesis of **30**, the piperazine moiety was attached as the last step by reacting commercially available unprotected 4,7-diazaspiro[2.5]octane with **189** (Scheme 26). This reaction occurred with a low yield, and so did the entire synthetic pathway (about 5% overall) [132]. Later, two optimized routes were patented [134,135]. In the first one, *t*-butyl 4,7-diazaspiro[2.5]octane-4-carboxylate was coupled with 2-nitro-5-bromopyridine, yielding **190**; the nitro group was hydrogenated using platinum on charcoal as a catalyst with a percentage of vanadium to avoid the formation of partially reduced intermediates [136]. Compound **191** was condensed with di-*t*-butyl malonate to build the pyrido[1,2-*a*]pyrimidin-4-one ring, and the 2-hydroxy group was esterified with tosyl chloride. Suzuki coupling with **194**, followed by the removal of the Boc-protecting group, gave Risdiplam with a yield of about 40%. In the final route, the yield was further improved. Compound **191** was reacted with Meldrum’s acid derivative **195**; the addition product **196** was boiled in *n*-propanol, resulting in the formation of the pyrimidine ring. Acid **197** was heated in *n*-propanol containing HCl to perform decarboxylation and deprotection, obtaining **30** in about a 60% yield.

Sotorasib (**31**) and Adagrasib (**32**) are KRAS^G12C^ inhibitors approved for the treatment of NSCLC. Kirsten rat sarcoma viral oncogene homolog (KRAS) is an oncogene frequently mutated in various types of cancer; the protein was considered undruggable until the crystal structure of the G12C mutant was solved, revealing a previously unknown pocket suitable for small molecule binding [137]. Sotorasib and Adagrasib carry an acryloyl residue that reacts with the nucleophilic C12 side chain, forming a covalent bond.

**Scheme 27:**
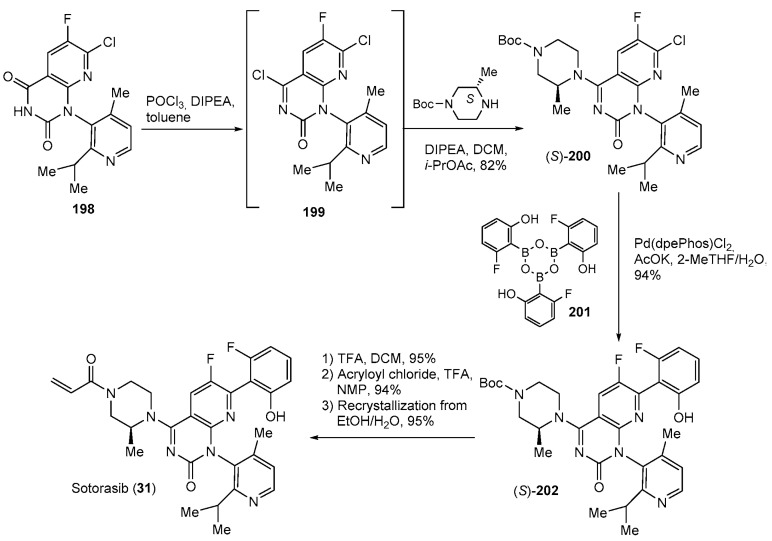
Synthesis of Sotorasib (**31**).

In both compounds, the piperazine ring is decorated with a *C*-substituent, a methyl group for **31** and a cyanomethyl group on **32**. The commercial route used to prepare **31** (Scheme 27) [138] is an optimization of the first published method [139], with the overall yield of the five-step process improved from 38 to 65%. The synthesis of the starting material **198** in its enantiomeric form (M-**198**) was reported in ref. [140]. The treatment of quinazolindione **198** with phosphorous oxychloride gave the chloro derivative **199**; without being isolated, it was reacted with commercially available *t*-butyl (*S*)-3-methylpiperazine-1-carboxylate, obtaining **200**. Pd-catalyzed Suzuki−Miyaura cross-coupling using boroxine **201** led to the piperazine derivative **202**. Deprotection, a reaction with acryloyl chloride, and recrystallization from ethanol-water gave **31**.

**Scheme 28:**
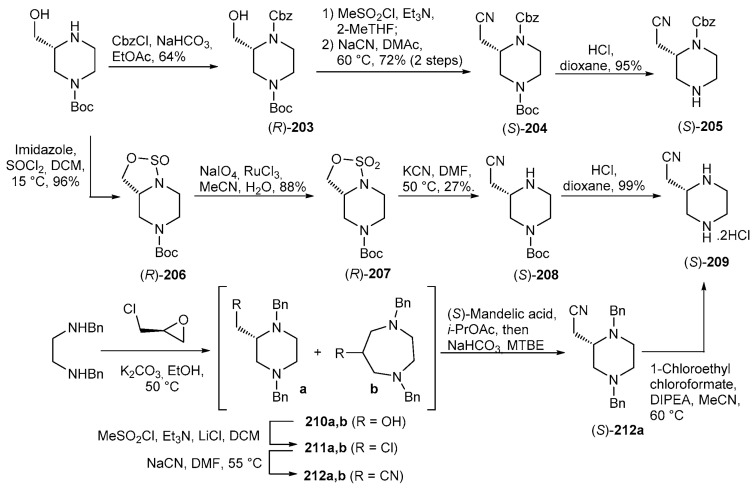
Synthesis of the piperazine building blocks of Adagrasib (**32**).

The synthesis of Adagrasib involves the use of (*S*)-2-(piperazin-2-yl)acetonitrile, which is not commercially available. Mirati Therapeutics described different syntheses (Scheme 28) for this building block as a *N*^1^-Cbz derivative ((*S*)-**205**) or as dihydrochloride salt ((*S*)-**209**) [141,142,143]. The preparation of compound (*S*)-**205** started with the protection of the NH group of *t*-butyl (*R*)-3-(hydroxymethyl)piperazine-1-carboxylate as a Cbz-derivative. The reaction of (*R*)-**203** with mesyl chloride, followed by treatment with NaCN, gave the protected piperazine (*S*)-**204**. Removal of the Boc protective group afforded (*S*)-**205** in a 44% yield. Compound (*S*)-**209** was prepared using the same starting molecule. A reaction with thionyl chloride afforded the oxathiazole oxide (*R*)-**206**, which, after oxidation to (*R*)-**207**, was reacted with potassium cyanide. Removal of the protective group of (*S*)-**208** under acidic conditions gave the desired piperazine (*S*)-**209** as dihydrochloride salt in a 23% yield. For the commercial supply, a different procedure was applied. The reaction of *N*,*N*^1^-dibenzylethanediamine with (*S*)-epichlorohydrin afforded (*R*)-(1,4-dibenzylpiperazin-2-yl)methanol **210a**, containing some 1,4-dibenzyl-1,4-diazepan-6-ol **210b**. The mixture was not separated; it was transformed into chloride **211a**,**b** by a reaction with mesylchloride and lithium chloride and then into cyanide **212a**,**b** by a reaction with sodium cyanide in DMF. The desired (*S*)-**212a** was purified from the mixture as (*S*)-mandelate salt, which was converted into the free base under basic conditions. The protective benzyl groups were finally removed using 1-chloroethyl chloroformate and *N,N*-diisopropylethylamine (DIPEA), affording (*S*)-**209** in an 88% yield.

**Scheme 29:**
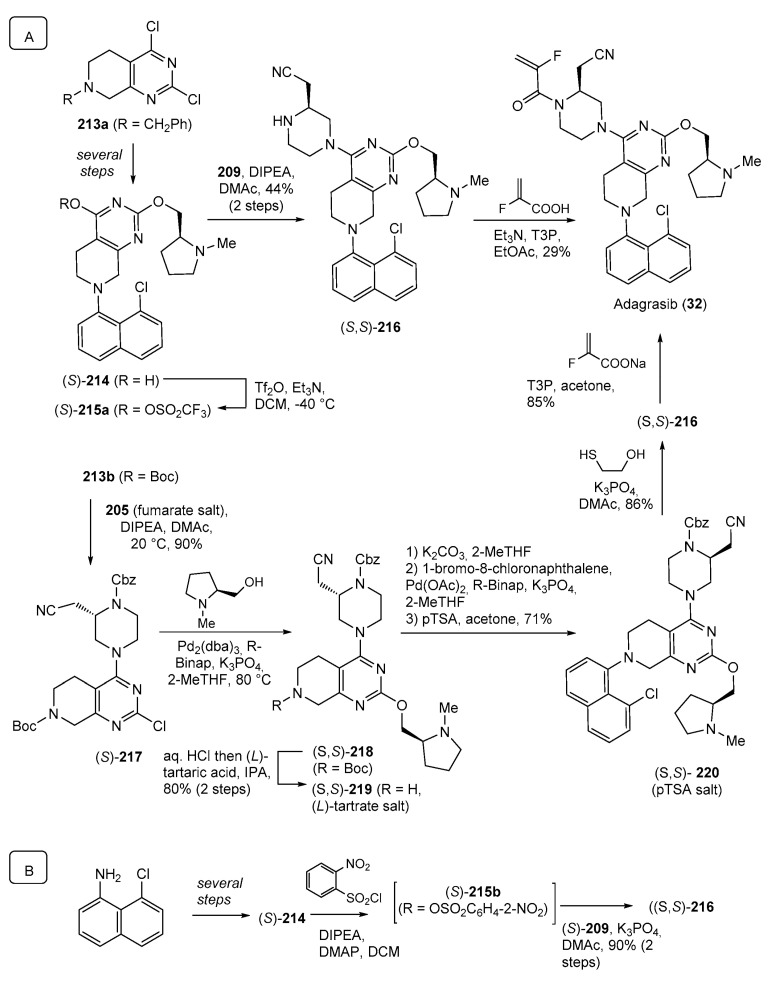
Synthesis of Adagrasib (**32**) from **213a** and **213b** (**A**); Synthesis of intermediate (*S*,*S*)-**216** from 8-chloronaphthalen-1-amine (**B**).

The synthesis of Adagrasib continued as shown in Scheme 29. The first published synthesis [142] started with tetrahydropyrido[3,4-d]pyrimidine **213a** (Scheme 29A, upper part), which was transformed into (*S*)-**214**. The piperazine moiety was inserted in the penultimate step by reacting compound (*S*)-**209** with triflate (*S*)-**215a**, in turn obtained by treating phenol (*S*)-**214** with triflic anhydride. The amidation of (*SS*)-**216** was then performed using 2-fluoroacrylic acid in the presence of a base (triethylamine) and of propanephosphonic anhydride (T3P) as an activating agent. This synthetic route yielded **32** with an overall yield of about 1%, starting with **213a**. To support the clinical studies and the initial commercial supply, another route was developed (Scheme 29A, bottom) [143], in which the piperazine group was inserted earlier. The reaction of the Boc-protected **213b** with (*S*)-**205** (as fumarate salt) led to (*S*)-**217** with a high yield and complete regioselectivity. The prolinol moiety was inserted by means of Pd-catalyzed C−O bond formation, and the Boc deprotection of (*S,S*)-**218** was achieved under acidic conditions, isolating (*S,S*)-**219** as *L*-tartrate salt. The naphthyl group was inserted by means of the Buchwald−Hartwig amination reaction; (*S,S*)-**220** was purified as tosylate salt. Removal of the Cbz group was accomplished using 2-mercapto-1-ethanol, and the amidation of (*S,S*)-**216** was performed using the previously employed activating agent (T3P) and sodium 2-fluoroacrylate. The sodium salt was used to minimize the decomposition of the acid, avoiding the addition of a base and improving the yield of this last step to 89% (70% after crystallization). The new process gave Adagrasib with an overall yield of 32%.

However, some criticisms for this route are related to the total cost due to the use of expensive Pd reagents, the early introduction of the costly chiral piperazine and the need to apply several protection/deprotection steps; therefore, a new process was developed (Scheme 29B) [144]. Intermediate (*S*)-**214** was prepared starting with 8-chloronaphthalen-1-amine to build first the piperidine ring and then the pyrimidine one, and then the prolinol appendage was attached via an SN_Ar_ reaction. The chiral piperazine was inserted through another SN_Ar_ reaction. The treatment of (*S*)-**209** with the 2-nitrobenzenesulfonyl ester (*S*)-**215b** and DIPEA avoided the formation of by-products deriving from nucleophilic attacks at the sulfur atom of the sulfonate group. The amidation step was performed as before without the need for final crystallization, owing to the high quality of the obtained material. This synthetic route did not involve the use of transition metals or protective groups. The five linear steps gave **32** in a 45% yield.

Fezolinetant (**33**) is an NK3 antagonist approved for the treatment of moderate to severe hot flashes caused by menopause. By inhibiting the binding of neurokinin B to NK3 receptors, Fezolinetant blocks the hypothalamic pituitary gonadal (HPG) axis, being effective in the treatment of sex hormone disorders [145]. In **33**, the piperazine ring is inserted into a [1,2,4]triazolo[4,3-a]pyrazine moiety; the stereogenic “magic methyl” group in position 8 is important, as it influences the amide orientation in the bioactive conformation, thus improving the activity with respect to the unsubstituted analog [146].

**Scheme 30:**
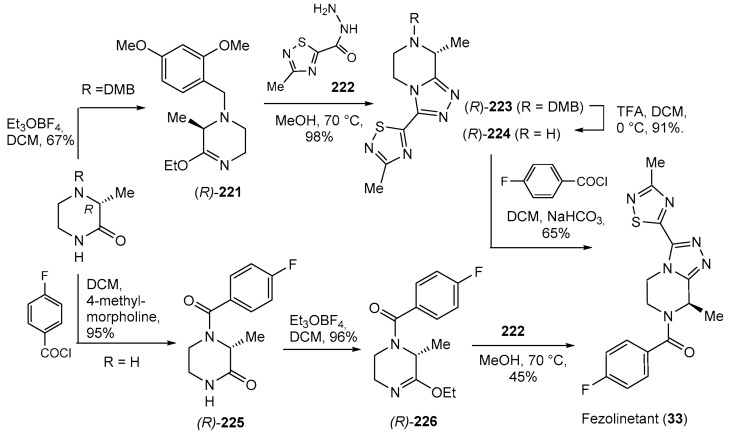
Synthesis of Fezolinetant (**33**).

The synthesis of **33** started with commercially available (*R*)-4-(2,4-dimethoxybenzyl)-3-methylpiperazin-2-one (Scheme 30), which was transformed into the piperazinoimidate **221** using Meerwein conditions (triethyloxonium tetrafluoroborate and sodium carbonate), and it treated with 3-methyl-1,2,4-thiadiazole-5-carbohydrazide **222** to build the triazole ring. The deprotection of **223** under acidic conditions and the acylation of the secondary amine **224** furnished **33**. The dimethoxybenzyl moiety was employed since racemization problems were encountered when using a different protective group (i.e., Boc). Moreover, the presence of the 2,4-dimethoxybenzyl (DMB) group shortened the reaction time in the condensation reaction [146,147]. Later, a patent reported that the insertion of the benzoyl moiety as an initial step could also afford the desired compound with minimal racemization. Thus, commercially available (*R*)-3-methylpiperazinone was treated with 4-fluorobenzoyl chloride; amide **225** was transformed into imidate **226**, which was treated with hydrazide **222** using 4-methylmorpholine as a base, obtaining **33**. This second route was faster and avoided the use of protective groups; however, the two methods gave similar yields (about 40%) [148].

Lumateperone (**34**) is an atypical antipsychotic used to manage both positive and negative symptoms in patients with schizophrenia. Lumateperone is able to directly modulate serotoninergic and dopaminergic transmission and indirectly modulates the glutamatergic system. It shows subnanomolar affinity for 5-HT2A receptors and interacts with nanomolar affinity with the D2 receptor and the serotonin transporter. Cellular assays revealed antagonistic properties on both receptors and SERT [149].

**Scheme 31:**
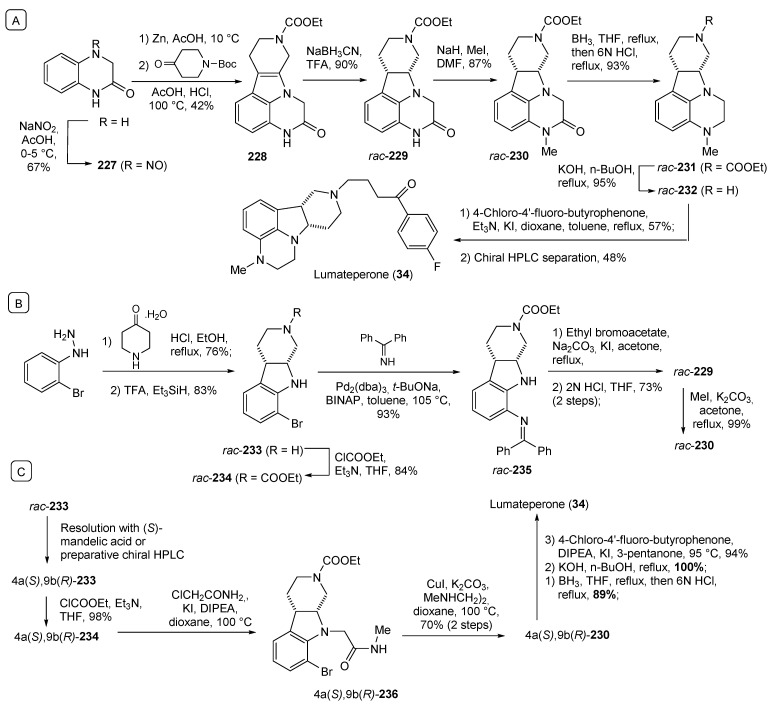
Synthesis of Lumateperone (**34**) from 3,4-dihydroquinoxalin-2(1H)-one (**A**), from (2-bromophenyl)hydrazine (**B**) and from 4a(*S*),9b(*R*)-**233** (**C**).

In **34**, the piperazine ring is inserted into a tetracyclic pyrido[4’,3’:4,5]pyrrolo[1,2,3-*de*]quinoxaline. Three different syntheses have been published for this compound [150,151]. In the first one (Scheme 31A), the starting material (3,4-dihydroquinoxalin-2(1*H*)-one) already contained a piperazinone ring. Treatment of this compound with sodium nitrite gave the nitroso derivative **227**. Reduction using zinc in acetic acid and the condensation of the resulting hydrazine derivative with ethyl 4-oxopiperidine-1-carboxylate gave the tetracyclic carbamate **228**, which was reduced on the indole double bond using sodium cyanoborohydride, obtaining *rac-***229**. Alkylation of the secondary amide nitrogen atom, followed by the selective reduction of the amide moiety of *rac-***230**, gave amine *rac-***231**. After the hydrolysis of the carbamate group, the alkylation of **232** with 4-chloro-4′-fluoro-butyrophenone gave a racemic compound whose enantiomers were separated by chiral HPLC, obtaining **34** (4a*S*,9b*R* isomer). The second route (Scheme 31B) provided a faster way to obtain racemic **230**. The indole ring was first built by condensing 2-bromophenyl hydrazine with 4-piperidone, and the indole double bond was reduced using triethylsilane and trifluoroacetic acid, obtaining the racemic *cis* indoline **233**. After the protection of the piperidine amine as carbamate, a Buchwald−Hartwig cross-coupling reaction of *rac-***234** with benzophenone immine afforded *rac-***235**. The alkylation of the indoline NH with ethyl bromoacetate, followed by the acidic hydrolysis of the immine, led to the formation of the piperazinone ring. After the methylation of the amide moiety, *rac-***230** was obtained and transformed into the final compound as before.

Later, a patent reported another route (Scheme 31C) in which the enantiomeric separation was performed in an earlier step on compound **233** by means of fractional crystallization of the mandelate salts or by means of chiral preparative HPLC [151]. In both cases, a high enantiomeric excess was obtained (yields were not indicated). After the usual protection of the piperidine NH group as ethyl carbamate (4a*S*,9b*R* **234**), the indoline NH moiety was alkylated using *N*-methyl-2-chloroacetamide, and on intermediate **236**, the closure of the piperazinone ring was achieved using a Cu-catalyzed Ullmann–Goldberg reaction. The reduction of 4a(*S*),9b(*R*)-**230** and the removal of carbamate was performed as before, and the final alkylation was performed using DIPEA in 3-pentanone, with a substantial improvement in the yield.

Fosdenopterin (**35**) is a molybdenum cofactor precursor used to reduce the risk of mortality in patients with molybdenum cofactor deficiency type A. Mutations of the molybdenum cofactor synthesis 1 gene reduce the endogenous availability of the cyclic pyranopterin monophosphate **35**, causing reduced molybdenum cofactor production. Fosdenopterin is administered as a supplement to overcome the cofactor deficiency [152].

**Scheme 32:**
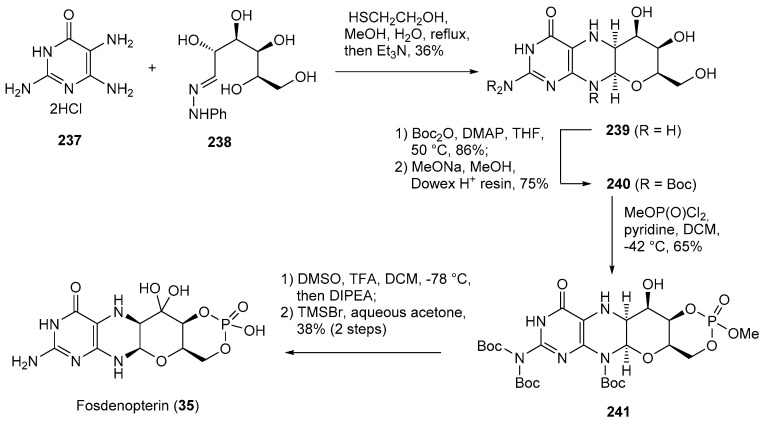
Synthesis of Fosdenopterin (**35**).

In **35**, the piperazine ring is inserted into a pyranopterin structure, with the two nitrogen atoms not carrying substituents. Such a heterocyclic structure was built starting with 2,5,6-triamino-3,4-dihydropyrimidin-4-one **237** and D-galactose phenylhydrazone **238** by means of a Viscontini reaction (Scheme 32) [153]. The treatment of **239** with an excess of Boc anhydride resulted in the formation of a mixture of hepta- and hexa-Boc-protected derivatives whose carbonate groups were hydrolyzed via the addition of sodium methoxide to achieve selective cleavage with respect to carbamates. Cyclic phosphate ester **241** was obtained after the treatment of **240** with methyl dichlorophosphate. Swern oxidation of the C-6 secondary alcohol gave the corresponding ketone, which was not isolated due to instability but was treated with trimethylbromosilane (TMSBr), achieving the removal of all protective groups (carbamates and ester) and furnishing **35**.

Trabectedin (**37**) is a natural compound derived from the Caribbean Sea squirt *Ecteinascidia Turbinata*. Lurbinectedin (**36**) is a synthetic derivative. Both compounds are DNA minor groove binders approved as anticancer drugs for different malignancies (soft tissue sarcomas and ovarian cancer for **37** and metastatic SCLC for **36**) and are presently used in clinical trials for other kinds of tumors [154]. Both compounds irreversibly bind DNA through the reaction of the aminoemiacetal functionality with the exocyclic guanine NH_2_ group in guanine-cytosine-rich sequences, as demonstrated for **37** [155].

**Scheme 33:**
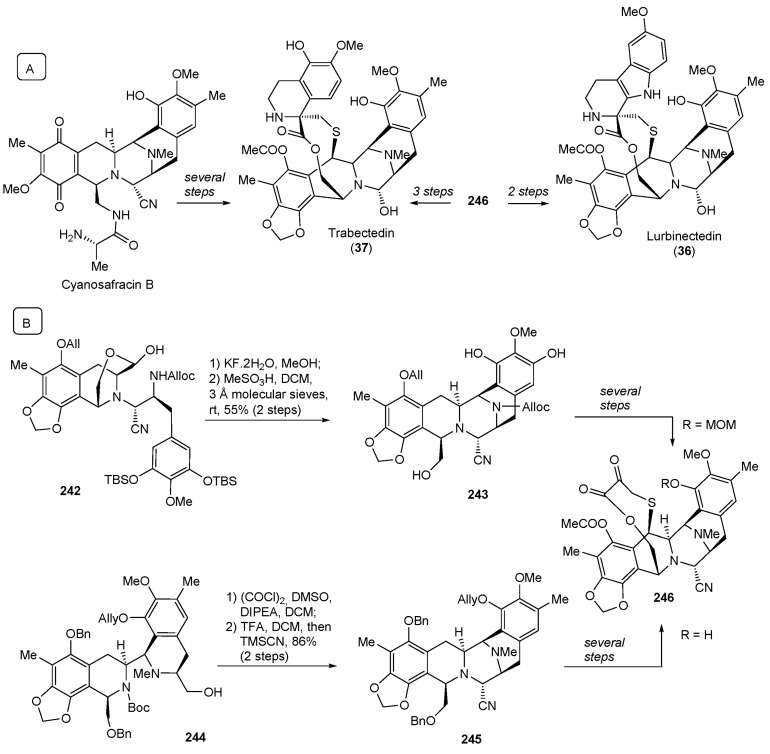
(**A**): Synthesis of Lurbinectedin (**36**) and Trabectedin (**37**); (**B**) Synthesis of lactone **246**. TBS = *tert*-butyldimethylsilyl; All = allyl; Alloc = COOAllyl.

Since the natural availability of **37** is limited, these molecules are produced via chemical synthesis. The compounds contain two tetrahydroisoquinoline moieties whose N-atoms are also part of a piperazine ring. Several total syntheses of **37** have been reported in the literature (see [156] and references cited therein), in which the pentacyclic core is assembled first, followed by the 10-membered lactone moiety. Since a discussion of the various routes is out of the scope of this review, we took into consideration only two of them, limiting the analysis to the building of the piperazine ring. The industrial method to produce Trabectedin and Lurbinectedin starts with Cyanosafracin B (Scheme 33A), already containing the pentacyclic core of these compounds [157]. In the historical route developed by Corey in 1996, the piperazine ring was assembled through an internal Mannich reaction between the carbamate *N*-atom and the lactol group of **242** after the removal of the *t*-butyldimethylsilyl groups (Scheme 33B). In this way, the piperazine and the second tetrahydroisoquinoline rings were formed at the same time, obtaining **243** [158]. In 2019, researchers from the Chinese Academy of Sciences developed a new route on a multigram scale, claimed to be efficient and scalable [156]. In this method, the piperazine ring was assembled starting with **244**. Swern oxidation of the hydroxymethyl group to aldehyde, *N*-Boc removal and an intramolecular Strecker reaction gave piperazine **245** in a good yield (Scheme 33B). Both **243** and **245** were then converted into lactone **246** and then transformed into the final compounds **36** and **37**.

Dolutegravir (**38**), Bictegravir (**39**) and Cabotegravir (**40**) are integrase inhibitors approved for the treatment of HIV infection. These compounds share the common *N*-benzyl-9-hydroxy-1,8-dioxo-1,3,4,8-tetrahydro-2*H*-pyrido[1,2-*a*]pyrazine-7-carboxamide structure, and they differ in the number of fluorine atoms on the benzyl carboxyamide group in 7 and in the size and absolute configuration of the oxygenated heterocycle fused to the N2-C3 bond. The incorporation of the amidic CO into a piperazinone ring facilitates the optimal orientation of this group for chelating Mg^2+^ ions in the active site, and the saturated *N*,*O*-heterocycle is important for the inhibitory activity against the Q148K mutated enzyme [159]. Dolutegravir and Bictegravir are formulated as tablets for daily administration, whereas Cabotegravir is formulated as a long-acting injectable suspension to be administered monthly or bi-monthly [160].

**Scheme 34:**
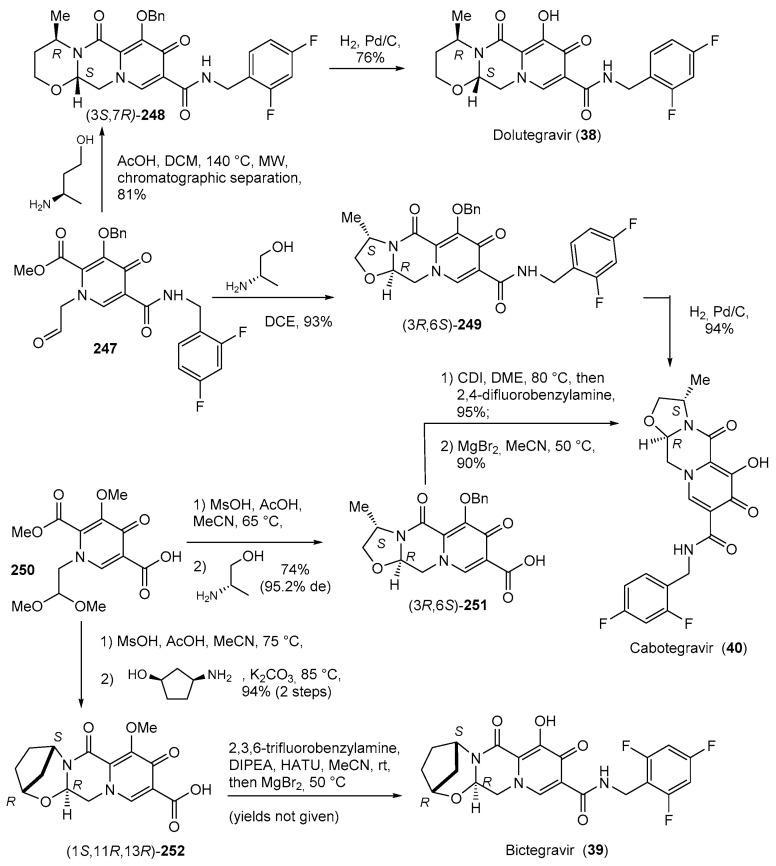
Synthesis of integrase inhibitors **38**–**40**.

Compounds **38**–**40** have the same central core and are prepared using similar pathways (Scheme 34) [159]. Compounds **38** and **40**, developed at GlaxoSmithKline, were prepared from carboxyamide **247** via a reaction with a suitable amino-alcohol to close the hemiaminal ring. Removal of the benzyl protective group by means of catalytic hydrogenation on **248** and **249** then yielded the desired compounds [161]. In the initial syntheses, pyridine **247** was prepared starting with maltol through several synthetic steps (not shown in Scheme 34); later, a faster method was developed starting with methyl 4-methoxy-3-oxobutanoate, which led to the 5-methoxy derivative **250**. This intermediate was reacted with (*S*)-2-aminopropanol, obtaining piperazinone **251**. Treatment with 2,4-difluorobenzylamine and CDI gave the corresponding carboxyamide, on which demethylation was accomplished with magnesium bromide, obtaining **40**. A similar pathway was also applied to the synthesis of **38** using (*R*)-3-aminobutanol.

Compound **250** was also used by Gilead in the synthesis of **39 [162]**. The reaction with (1*R*,3*S*)-3-amino cyclopentan-1-ol gave hemiaminal **252**, which was transformed into the final compound **39** using the same method seen before.

## 4. Role of the Piperazine Moiety

Forty new small molecules carrying a piperazine ring were approved by the FDA between January 2011 and June 2023. Among them, the largest therapeutic class is represented by kinase inhibitors (**1**, **2**, **4**, **6**–**8**, **18**–**23**, **29**, **31** and **32**), developed to treat different types of cancer, even if one of them (Nintedanib, **20**) was approved for a different indication (treatment of idiopathic pulmonary fibrosis). The second largest group of piperazine derivatives consists of central nervous system (CNS) receptors modulators (**3**, **10**, **11**, **13**–**17**, **26**, **33** and **34**). The piperazine is indeed a recurrent motif in these two therapeutic classes [4].

The role of the piperazine moiety in compounds **1**–**40** is various. For some compounds, it is related to the modulation of the physicochemical properties, such as basicity and solubility, which positively affect pharmacokinetics. This has been reported for Entrectinib **8** [47], Bosutinib **18** [93], Ponatinib **19** [96], Brigatinib **23** [106] and Olaparib (**27**) [120]. Additionally, the protonated piperazine *N*-atom may contribute to the interaction with the target macromolecule, as is suggested for the kinase inhibitors Palbociclib **1**, Ribociclib **2**, Abemaciclib **21** [11] and Nintedanib **20** [97] and the RNA slicing modifier Risdiplam **30** [132]. A particular example is Maralixibat **24**, whose piperazine ring is included in a DABCO structure; the quaternary N-atom limits absorption, allowing the pharmacological activity to be carried out from the ileal lumen [99]. Moreover, the insertion of DABCO was one of the modifications leading to a crystalline and non-hygroscopic compound [163]. In the case of Venetoclax **12**, the piperazine moiety was inserted to increase polarity in specific parts of the molecule in order to limit the interaction with plasma proteins [164].

In some other instances, the insertion of a piperazine moiety has improved safety. For Infigratinib (**7**), the *N*-ethylpiperazine group was chosen among other basic groups because it prevented the inhibition of cytochrome P450 isoforms [45]. For Zavegepant (**26**), replacing (*N*-methylpiperidinyl)piperidine with (*N*-methylpiperidinil)piperazine reduced nasal irritation after the administration of the drug [118].

Regarding the CNS receptor modulators **3**, **13**–**17** and **34**, which act on dopaminergic and/or serotoninergic systems, the arylpiperazine moiety is part of the pharmacophore. This structural motif allows interaction at the orthosteric site of these receptors, since the *N*^1^-aryl moiety is inserted in a hydrophobic pocked formed by aromatic residues and is located close to the aspartate residue, which establish the pivotal ion–ion interaction with the basic (and protonated) piperazine N^4^-atom [165,166,167].

Some drugs contain the piperazine ring because it was already present in the lead (hit) in the discovery campaign (**25** [111], **28** [126], **31** [168] and **32** [142]), and it was conserved in the final molecule. For some other compounds, information about their design and optimization have not yet been reported in the literature, so the role of the piperazine moiety cannot be properly evaluated.

## 5. Conclusions

The analysis of the methods used to prepare compounds **1**–**40** shows that most synthetic routes use a synthon that already contains the piperazine ring (compounds **1**–**13**, **15**–**31**). As said in the introduction, many useful *N*-alkyl, *N*-acyl or *N*-protected piperazines are commercially available, often at low costs. The building of the piperazine (compounds **14**, **32** and **34**–**37**) or piperazinone ring (**6**, **38**–**40**) is necessary or suitable for only a few compounds.

Regarding the reaction applied to the synthesis of *N*-arylpiperazines, Pd-catalyzed Buchwald–Hartwig coupling is more often used in discovery chemistry than in process chemistry due to the concern regarding the use of large quantities of expensive Pd catalysts, whose complete removal from the mixtures has been demonstrated in some cases to be difficult. Safety and environmental concerns regarding the use of Cu catalysts likely also limits the use of the Ullmann–Goldberg reaction. The SN_Ar_ reaction is most often utilized in process chemistry due to the ease of work-up and the purification of the reaction mixtures. Venetoclax **12** is an exception, because the original SN_Ar_ reaction is replaced with a Pd-catalyzed amination reaction. In this way, the whole process is improved by reducing the problems connected to the handling of an unstable intermediate (**104**) and by exploiting a cleaner preparation of a starting compound (**109**).

To prepare *N*-alkyl derivatives, the reaction of piperazine reactants with alkyl bromides or chlorides is often helped by the addition of sodium, potassium or ammonium iodide to improve the yield and to avoid the formation of by-products that complicate the purification of the drug. Only in one case, an alkyl mesilate is used (**17**). When reductive amination is applied, sodium triacethoxyborohydride is employed in the preparation of compounds **12**, **17**, **21** and **25**, and it is formed in situ from sodium cyanoborohydride and acetic acid in the synthesis of **23**. It is of note that, in the synthesis of **21**, sodium triacethoxyborohydride works well for the reductive amination of 6-bromo-nicotinaldehyde (99% yield), but in the case of the less reactive nicotinic derivative **153**, the reaction is not complete, leading to the application of a different method (Leuckart–Wallach reaction, using formic acid).

Ab initio synthesis of the piperazine ring is performed for *N*-aryl derivatives **14** and **16** starting with an aniline precursor and bis(2-chloroethyl)ammine or iethanolamine, respectively, and for the Adagrasib building block **209**, it is performed by condensing dibenzylethanediamine and epichloridrine. The synthesis of **33** and **34** involves the preparation and/or functionalization of piperazin-2-one derivatives, whereas the complex compounds **35**–**37** require ad hoc methods. Finally, the piperazinone rings of **6** and **38**–**40** are easily formed via the intramolecular cyclization of amine and ester group intermediates.

In general, synthetic methods are optimized through the careful control of the reaction conditions regarding solvents, reagents, temperatures and work-up procedures.

## Figures and Tables

**Table 1:** New small molecules approved by FDA between January 2011 and June 2023, containing a piperazine ring with substituents only on the *N*-atoms.

Compound Number	Name, Year of Approval, Mechanism of Action and Therapeutic Indication	Structure
**1**	Palbociclib (2015): Cyclin-Dependent Kinase 4/6 inhibitor (treatment of metastatic breast cancer)	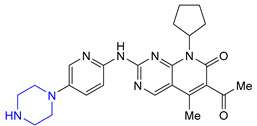
**2**	Ribociclib (2017): Cyclin-Dependent Kinase 4/6 inhibitor (treatment of metastatic breast cancer)	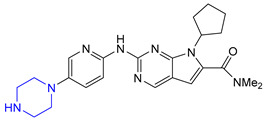
**3**	Vortioxetine (2013): serotoninergic modulator (treatment of major depressive disorder)	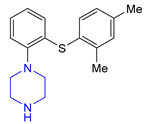
**4**	Avapritinib (2020): platelet-derived growth factor receptor alpha inhibitor (treatment of Gastrointestinal Stromal Tumor)	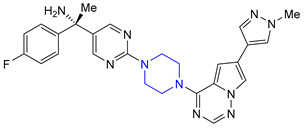
**5**	Letermovir (2017): cytomegalovirus DNA terminase inhibitor (to prevent infection in bone marrow transplant)	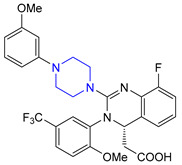
**6**	Trilaciclib (2021): Cyclin-Dependent Kinase 4/6 inhibitor (mitigation of chemotherapy-induced myelosuppression in small cell lung cancer)	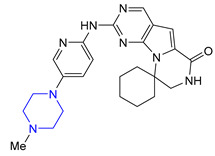
**7**	Infigratinib (2021): fibroblast growth factor receptor inhibitor (treatment of cholangiocarcinoma)	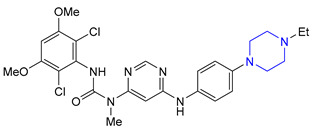
**8**	Entrectinib (2019): ALK, ROS1 and Trk kinase inhibitor (treatment of metastatic non-small cell lung cancer)	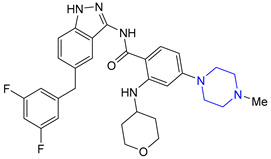
**9**	Avatrombopag (2018): thrombopoietin receptor agonist (treatment of thrombocytopenia)	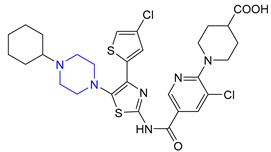
**10** **11**	Netupitant (2014) and Fosnetupitant (2018): NK1 receptor antagonists (treatment of nausea and vomiting in patients undergoing cancer chemotherapy, in combination with palosetron)	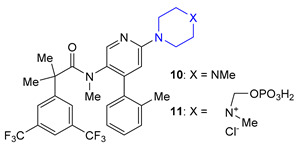
**12**	Venetoclax (2016): Bcl-2 blocker (treatment of chronic lymphocytic leukemia in patients with a specific chromosomal abnormality)	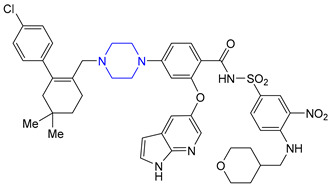
**13**	Brexpiprazole (2015): (treatment of schizophrenia and major depressive disorder)	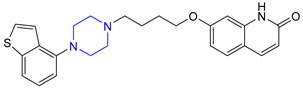
**14**	Vilazodone (2011): serotoninergic modulator (treatment of major depressive disorder)	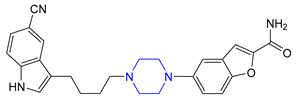
**15**	Flibanserin (2015): 5-HT1A agonist (treatment of acquired, generalized hypoactive sexual desire disorder in premenopausal women)	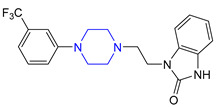
**16**	Aripiprazole Lauroxil (2015): long-acting antipsychotic (treatment of schizophrenia)	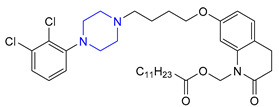
**17**	Cariprazine (2015): D2/D3 receptors partial agonist (treatment of schizophrenia and bipolar disorder in adults)	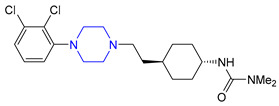
**18**	Bosutinib (2012): Bcr-Abl tyrosine-kinase inhibitor (treatment of chronic myelogenous leukemia)	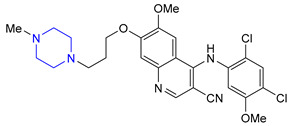
**19**	Ponatinib (2012): Bcr-Abl tyrosine-kinase inhibitor (treatment of chronic myeloid leukemia and Philadelphia chromosome positive acute lymphoblastic leukemia)	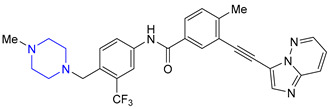
**20**	Nintedanib (2014): receptor and non-receptor tyrosine kinase inhibitor (treatment of idiopathic pulmonary fibrosis)	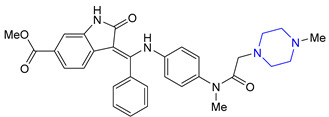
**21**	Abemaciclib (2017): Cyclin-Dependent Kinase 4/6 inhibitor (treatment of metastatic breast cancer)	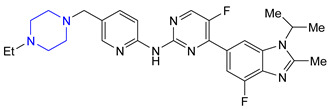
**22**	Gilteritinib (2018): FMS-like tyrosine kinase 3 inhibitor (treatment of Acute Myeloid Leukemia)	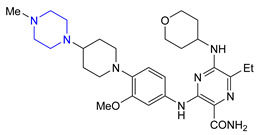
**23**	Brigatinib (2017): anaplastic lymphoma kinase/epidermal growth factor receptor inhibitor (treatment of non-small cell lung cancer)	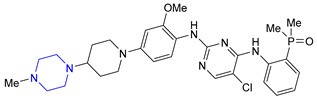
**24**	Maralixibat (2021): ileal bile acid transporter inhibitor (treatment of cholestatic pruritus associated with Alagille syndrome)	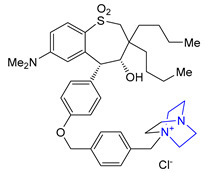
**25**	Mitapivat (2022): pyruvate kinase activator (treatment of hemolytic anemia in pyruvate kinase deficiency)	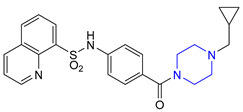
**26**	Zavegepant (2023): calcitonin gene-related peptide receptor antagonist (treatment of migraines)	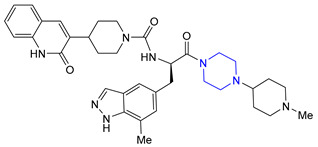
**27**	Olaparib (2014): poly ADP ribose polymerase inhibitor (treatment of advanced ovarian cancer)	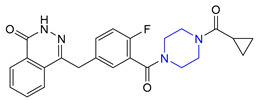
**28**	Fostemsavir (2020): HIV attachment inhibitor (treatment of HIV infection)	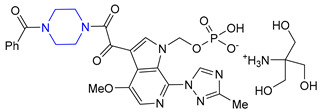

**Table 2:** New small molecules approved by FDA between January 2011 and June 2023, containing a piperazine moiety with substituents in the ring *C*-atoms or inserted into a polycyclic structure.

Compound Number	Name, Year of Approval, Mechanism of Action and Therapeutic Indication	Structure
**29**	Selpercatinib (2020): Rearranged during Transfection (RET) inhibitor (treatment of lung and thyroid cancers)	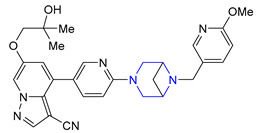
**30**	Risdiplam (2020): Survival Motor Neuron-2 RNA splicing modifier (treatment of spinal muscular atrophy)	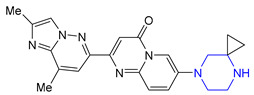
**31**	Sotorasib (2021): KRAS^G12C^ inhibitor (treatment of non-small cell lung cancer)	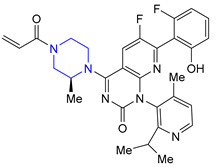
**32**	Adagrasib (2022): KRAS^G12C^ inhibitor (treatment of locally advanced or metastatic non-small cell lung cancer)	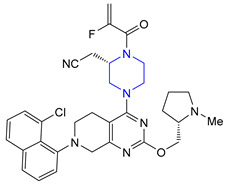
**33**	Fezolinetant (2023): NK3 receptor antagonist (treatment of moderate to severe hot flashes caused by menopause)	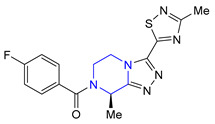
**34**	Lumateperone (2019): 5-HT2A/D2 antagonist (schizophrenia)	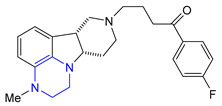
**35**	Fosdenopterin (2021): molybdenum cofactor precursor (to reduce the risk of mortality in patients with molybdenum cofactor deficiency Type A)	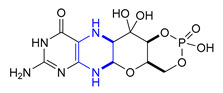
**36**	Lurbinectedin (2020): DNA minor groove binder (treatment of metastatic small cell lung cancer)	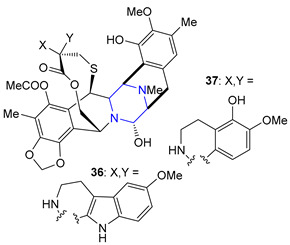
**37**	Trabectedin (2015): DNA minor groove binder (treatment of specific soft tissue sarcomas: liposarcoma and leiomyosarcoma)	
**38**	Dolutegravir (2013): Integrase inhibitor (treatment of HIV infection)	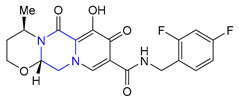
**39**	Bictegravir (2018): Integrase inhibitor, approved in combination with emtricitabine and tenofovir alafenamide (treatment of HIV infection)	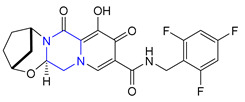
**40**	Cabotegravir (2021): Integrase inhibitor, approved in combination with rilpivirine (treatment of HIV infection)	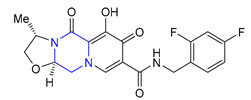

## Abbreviations

**5-HT**, serotonin; **ABC**, ATP-binding cassette; **ADP**, adenosine diphosphate; **AIBN**, 2,2′-azobisisobutyronitrile; **ALK**, anaplastic lymphoma kinase; **Amphos**, di-*tert*-butyl(4-dimethylaminophenyl)phosphine)-*N*, *N*-dimethylbenzenamide; **ATP**, adenosine triphosphate; **BCL**, B Cell Lymphoma protein; **BCRP**, breast cancer resistance protein; **BH3**, homology domain 3; **BINAP**, 2,2′-bis(diphenylphosphino)-1-1′-binaphthalene; **BRCA**, breast-related cancer antigens; **CDI**, 1,1′-carbonyldiimidazole; **CDK**, Cyclin-Dependent Kinase; **CGRP**, calcitonin gene-related peptide; **CML**, chronic myelogenous leukemia; **CMV**, cytomegalovirus; **CNS**, central nervous system; **D**, dopamine; **DABCO**, 1,4-diazabicyclo[2.2.2]octane; **DBU**, 1,8-diazabicyclo[5.4.0]undec-7-ene; **DCE**, 1,2-dichloroethane; **DCM**, dichloromethane; **DDQ**, 2,3-dichloro-5,6-dicyano-1,4-benzoquinone; **de**, diastereomeric excess; **DIPEA**, *N,N*-diisopropylethylamine; **DMAc**, N,N-dimethylacetamide; **DMAP**, 4-(*N,N*-dimethylamino)pyridine; **DMB**, 2,4-dimethoxybenzyl; **DME**, 1,2-dimethoxyethane; **DMF**, dimethylformamide; **DMSO**, dimethyl sulfoxide; **DNA**, deoxyribonucleic acid; **DPEPhos**, Bis[2-diphenylphosphino)phenyl]ether; **DPPCl**, diphenylphosphinic chloride; **EDC**, 1-ethyl-3-(3-dimethylaminopropyl)carbodiimide; **ee**, enantiomeric excess; **EtOAc**, ethyl acetate; **FDA**, Food and Drug Administration; **FGFR**, fibroblast growth factor receptor; **FLT3**, FMS-like tyrosine kinase gene; **HATU**, 1-[Bis(dimethylamino)methylene]-1*H*-1,2,3-triazolo[4,5-*b*]pyridinium 3-oxid hexafluorophosphate; **HBTU**, *N,N,N′,N′*-tetramethyl-(O-(1*H*-benzotriazol-1-yl)uronium hexafluorophosphate; **HIV**, human immunodeficiency virus; **HOBt**, 1-hydroxybenzotriazole; **HPLC**, high-performance liquid chromatography; **HTS**, high-throughput screening; **IBAT**, ileal bile acid transporter; **IPA**, isopropyl alcohol; ***i*PrOAC**, isopropyl acetate; *iv*, intravenous; **KRAS**, Kirsten rat sarcoma viral oncogene homolog; **LiAlH_4_**, lithium aluminum hydride; **LiHMDS**, lithium bis(trimethylsilyl)amide; **m-CPBA**, meta-chloroperoxybenzoic acid; **MDR1**, multidrug-resistant transporter 1; **MeCN**, acetonitrile; **MTBE**, ethyl *tert*-butyl ether; **MW**, microwave; **NBS**, *N*-bromosuccinimide; **NK**, neurokinin receptor; **NMP**, *N*-methylpyrrolidone; **NSCLC**, non-small cell lung cancer; **PARP**, poly-ADP-ribose-polymerase; **PDGFR**, platelet-derived growth factor receptor; **PDGFRA**, platelet-derived growth factor receptor alpha; **PET**, positron emission tomography; **PK**, pyruvate kinase; **PyBoP**, (benzotriazol-1-yloxy)tripyrrolidinophosphonium hexafluorophosphate; **RET**, rearranged during transfection; **RNA**, ribonucleic acid; **rt**, room temperature; **SCLC**, small cell lung cancer; **SERT**, serotonin transporter; **SMN**, survival of motor neuron; **SN_Ar_**, aromatic nucleophilic substitution; **STS**, soft tissue sarcomas; **T3P**, propanephosphonic anhydride; **TBAF**, tetrabutylammonium fluoride; **TFA**, trifluoroacetic acid; **THF**, tetrahydrofuran; **TMEDA**, *N*,*N*,*N′*,*N′*-tetramethyl-1,2-ethylenediamine; **TMSBr**, trimethylbromosilane; **TMSCHN_2_**, (Diazomethyl)trimethylsilane; **TMSCN**, trimethylsilyl cyanide; **TPO**, thrombopoietin; **TRK**, tropomyosin receptor kinase; **VEGFR**, vascular endothelial growth factor.

## References

[1] Taylor R.D., Maccoss M., Lawson A.D.G. (2014). Rings in Drugs. J. Med. Chem..

[2] Vitaku E., Smith D.T., Njardarson J.T. (2014). Analysis of the Structural Diversity, Substitution Patterns, and Frequency of Nitrogen Heterocycles among U.S. FDA Approved Pharmaceuticals. J. Med. Chem..

[3] Meanwell N.A., Loiseleur O. (2022). Applications of Isosteres of Piperazine in the Design of Biologically Active Compounds: Part 1. J. Agric. Food Chem..

[4] Romanelli M.N., Manetti D., Braconi L., Dei S., Gabellini A., Teodori E. (2022). The piperazine scaffold for novel drug discovery efforts: The evidence to date. Exp. Opin. Drug Discov..

[5] Dinsmore C.J., Beshore D.C. (2002). Syntheses and transformations of piperazinone rings. A review. Org. Prep. Proced. Int..

[6] Dömling A., Huang Y. (2010). Piperazine Scaffolds via Isocyanide-Based Multicomponent Reactions. Synthesis.

[7] Ye Z., Gettys K.E., Dai M. (2016). Opportunities and challenges for direct C–H functionalization of piperazines. Beilstein J. Org. Chem..

[8] Gettys K.E., Ye Z., Dai M. (2017). Recent Advances in Piperazine Synthesis. Synthesis.

[9] Seifinoferest B., Tanbakouchian A., Larijani B., Mahdavi M. (2021). Ullmann-Goldberg and Buchwald-Hartwig C−N Cross Couplings: Synthetic Methods to Pharmaceutically Potential N-Heterocycles. Asian J. Org. Chem..

[10] Bunnett J.F., Zahler R.E. (1951). Aromatic Nucleophilic Substitution Reactions. Chem. Rev..

[11] Chen P., Lee N.V., Hu W., Xu M., Ferre R.A., Lam H., Bergqvist S., Solowiej J., Diehl W., He Y.-A. (2016). Spectrum and Degree of CDK Drug Interactions Predicts Clinical Performance. Mol. Cancer Ther..

[12] Poratti M., Marzaro G. (2019). Third-generation CDK inhibitors: A review on the synthesis and binding modes of Palbociclib, Ribociclib and Abemaciclib. Eur. J. Med. Chem..

[13] Toogood P.L., Harvey P.J., Repine J.T., Sheehan D.J., Vanderwel S.N., Zhou H., Keller P.R., Mcnamara D.J., Sherry D., Zhu T. (2005). Discovery of a Potent and Selective Inhibitor of Cyclin-Dependent Kinase 4/6. J. Med. Chem..

[14] Calienni J.V., Chen G.-P., Gong B., Kapa P.K., Saxena V. (2012). Salt(S) of 7-Cyclopentyl-2-(5-Piperazin-1-Yl-Pyridin-2-Ylamino)-7h-Pyrrolo[2,3-d]Pyrimidine-6-Carboxylic Acid Dimethylamide and Processes of Making Thereof. U.S. Patent.

[15] Duan S., Place D., Perfect H.H., Ide N.D., Maloney M., Sutherland K., Price Wiglesworth K.E., Wang K., Olivier M., Kong F. (2016). Palbociclib Commercial Manufacturing Process Development. Part I: Control of Regioselectivity in a Grignard-Mediated SNAr Coupling. Org. Process Res. Dev..

[16] Chekal B.P., Ewers J., Guinness S.M., Ide N.D., Leeman K.R., Post R.J., Rane A.M., Sutherland K., Wang K., Webster M. (2016). Palbociclib Commercial Manufacturing Process Development. Part III. Deprotection Followed by Crystallization for API Particle Property Control. Org. Process Res. Dev..

[17] Besong G., Brain C.T., Brooks C.A., Congreve M.S., Dagostin C., He G., Hou Y., Howard S., Li Y., Lu Y. (2010). Pyrrolopyrimidine Compounds as CDK Inhibitors.

[18] Pellegatti L., Hafner A., Sedelmeier J. (2016). A Two-Step Continuous-Flow Procedure towards Ribociclib. J. Flow Chem..

[19] Tavares F.X., Strum J.C. (2013). CDK Inhibitors. U.S. Patent.

[20] Smith A., White H.S., Tavares F.X., Krasutsky S., Chen J.-X., Dorrow R.L., Zhong H. (2018). Synthesis of N-(Heteroaryl)-pyrrolo[3,2-d]pyrimidin-2-amines.

[21] Bang-Andersen B., Ruhland T., Jørgensen M., Smith G., Frederiksen K., Jensen K.G., Zhong H., Nielsen S.M., Hogg S., Mørk A. (2011). Discovery of 1-[2-(2,4-Dimethylphenylsulfanyl)phenyl]piperazine (Lu AA21004): A Novel Multimodal Compound for the Treatment of Major Depressive Disorder. J. Med. Chem..

[22] Sanchez C., Asin K.E., Artigas F. (2015). Vortioxetine, a novel antidepressant with multimodal activity: Review of preclinical and clinical data. Pharmacol. Ther..

[23] Bishop M.M., Fixen D.R., Linnebur S.A., Pearson S.M. (2021). Cognitive effects of vortioxetine in older adults: A systematic review. Ther. Adv. Psychopharmacol..

[24] Alcántara Montero A., Pacheco De Vasconcelos S.R. (2022). Role of vortioxetine in the treatment of neuropathic pain. Rev. Esp. Anestesiol. Reanim. (Engl. Ed.).

[25] Bang-Andersen B., Faldt A., Moerk A., Lopez De Diego H., Holm R., Stensboel T.B., Ringgaard L.M., Mealy M.J., Rock M.H., Brodersen J. (2005). 1-[2-(2,4-Dimethylphenylsulfanyl)-phenyl] Piperazine as a Compound with Combined Serotonin Reuptake, 5-HT3 and 5-HT1a Activity for the Treatment of Cognitive Impairment.

[26] Zupancic B. (2015). Synthesis of Vortioxetine via (2,4-Dimethylphenyl)(2-iodophenyl)sulfane Intermediate.

[27] Zupancic B. (2014). New Process for the Synthesis of 1-(2-((2,4-Dimethylphenyl)thio)phenyl)piperazine.

[28] Zupancic B., Sterk D., Maras N. (2015). Synthesis of Vortioxetine via (2-(Piperazine-1-yl)phenvl)lithium Intermediates.

[29] Zupancic B. (2014). New Process for the Synthesis of 1-(2-((2,4-Dimethylphenyl)thio)phenyl)piperazine.

[30] Dhillon S. (2020). Avapritinib: First Approval. Drugs.

[31] Klug L.R., Kent J.D., Heinrich M.C. (2018). Structural and clinical consequences of activation loop mutations in class III receptor tyrosine kinases. Pharmacol. Ther..

[32] Evans E.K., Gardino A.K., Kim J.L., Hodous B.L., Shutes A., Davis A., Zhu X.J., Schmidt-Kittler O., Wilson D., Wilson K. (2017). A precision therapy against cancers driven by *KIT/PDGFRA* mutations. Sci. Transl. Med..

[33] Wu C.-P., Lusvarghi S., Wang J.-C., Hsiao S.-H., Huang Y.-H., Hung T.-H., Ambudkar S.V. (2019). Avapritinib: A Selective Inhibitor of KIT and PDGFRα that Reverses ABCB1 and ABCG2-Mediated Multidrug Resistance in Cancer Cell Lines. Mol. Pharm..

[34] Hodous B.L., Kim J.L., Wilson K.J., Wilson D., Zhang Y. (2015). Compositions Useful for Treating Disorders Related to KIT. U.S. Patent.

[35] Waetzig J., Mar B., Heinrich B., Wilkie G., Maceachern L. (2020). Crystalline forms of (S)-1-(4-Fluorophenyl)-1-(2-(4-(6-(1-methyl-1h-pyrazol-4-yl)pyrrolo[2,1-f][1,2,4]triazin-4-yl)piperazinyl)-pyrimidin-5-yl)ethan-1-amine and Methods of Making.

[36] Porcs-Makkay M., Bertha F., Molnár E., Németh G., Horváth S., Szebelédi I., Bali B., Tellér M., Kátainé Fadgyas K. (2022). Process for Obtaining Avapritinib and Its Intermediates.

[37] Kim E.S. (2018). Letermovir: First Global Approval. Drugs.

[38] Lischka P., Hewlett G., Wunberg T., Baumeister J., Paulsen D., Goldner T., Ruebsamen-Schaeff H., Zimmermann H. (2010). In Vitro and In Vivo Activities of the Novel Anticytomegalovirus Compound AIC246. Antimicrob. Agents Chemother..

[39] Gentry B.G., Bogner E., Drach J.C. (2019). Targeting the terminase: An important step forward in the treatment and prophylaxis of human cytomegalovirus infections. Antivir. Res..

[40] Wunberg T., Baumeister J., Betz U., Jeske M., Lampe T., Nikolic S., Reefschlager J., Schohe-Loop R., Sussmeier F., Zimmermann H. (2007). Substituted Dihydroquinazolines.

[41] Paulus K., Schwab W., Grunder D., Van Hoogevest P. (2015). Pharmaceutical Composition Containing an Antivirally Active Dihydroquinazoline Derivative. U.S. Patent.

[42] Humphrey G.R., Dalby S.M., Andreani T., Xiang B., Luzung M.R., Song Z.J., Shevlin M., Christensen M., Belyk K.M., Tschaen D.M. (2016). Asymmetric Synthesis of Letermovir Using a Novel Phase-Transfer-Catalyzed Aza-Michael Reaction. Org. Process Res. Dev..

[43] Kang C. (2021). Infigratinib: First Approval. Drugs.

[44] Furet P., Caravatti G., Guagnano V., Lang M., Meyer T., Schoepfer J. (2008). Entry into a new class of protein kinase inhibitors by pseudo ring design. Bioorg. Med. Chem. Lett..

[45] Guagnano V., Furet P., Spanka C., Bordas V., Le Douget M., Stamm C., Brueggen J., Jensen M.R., Schnell C., Schmid H. (2011). Discovery of 3-(2,6-Dichloro-3,5-dimethoxy-phenyl)-1-{6-[4-(4-ethyl-piperazin-1-yl)-phenylamino]-pyrimidin-4-yl}-1-methyl-urea (NVP-BGJ398), A Potent and Selective Inhibitor of the Fibroblast Growth Factor Receptor Family of Receptor Tyrosine Kinase. J. Med. Chem..

[46] Jiang Q., Li M., Li H., Chen L. (2022). Entrectinib, a new multi-target inhibitor for cancer therapy. Biomed. Pharmacother..

[47] Menichincheri M., Ardini E., Magnaghi P., Avanzi N., Banfi P., Bossi R., Buffa L., Canevari G., Ceriani L., Colombo M. (2016). Discovery of Entrectinib: A New 3-Aminoindazole As a Potent Anaplastic Lymphoma Kinase (ALK), c-ros Oncogene 1 Kinase (ROS1), and Pan-Tropomyosin Receptor Kinases (Pan-TRKs) inhibitor. J. Med. Chem..

[48] Bandiera T., Lombardi B.A., Nesi M., Perrone E., Bossi R., Polucci P. (2008). Indazole Derivatives as Kinase Inhibitors for the Treatment of Cancer.

[49] Shirley M. (2018). Avatrombopag: First Global Approval. Drugs.

[50] Zhang J., Alt J., Yang M., Baranwal A., Luby T.M. (2008). AKR-501 Activates the Thrombopoietin Receptor through Interaction with the Transmembrane Domain. Blood.

[51] Kuter D.J. (2022). The structure, function, and clinical use of the thrombopoietin receptor agonist avatrombopag. Blood Rev..

[52] Sugasawa K., Watanuki S., Koga Y., Nagata H., Obitsu K., Wakayama R., Hirayama F., Suzuki K.-I. (2004). 2-Acylaminothiazole Derivative or Salt Thereof.

[53] Shirley M. (2021). Netupitant/Palonosetron: A Review in Chemotherapy-Induced Nausea and Vomiting. Drugs.

[54] Hoffmann T., Bös M., Stadler H., Schnider P., Hunkeler W., Godel T., Galley G., Ballard T.M., Higgins G.A., Poli S.M. (2006). Design and synthesis of a novel, achiral class of highly potent and selective, orally active neurokinin-1 receptor antagonists. Bioorg. Med. Chem. Lett..

[55] Boes M., Branca Q., Galley G., Godel T., Hoffmann T., Hunkeler W., Schnider P., Stadler H. (2001). 4-Phenyl-pyridine Derivatives. U.S. Patent.

[56] Hoffmann-Emery F., Hilpert H., Scalone M., Waldmeier P. (2006). Efficient Synthesis of Novel NK1 Receptor Antagonists:  Selective 1,4-Addition of Grignard Reagents to 6-Chloronicotinic Acid Derivatives. J. Org. Chem..

[57] Harrington P.J., Johnston D., Moorlag H., Wong J.-W., Hodges L.M., Harris L., Mcewen G.K., Smallwood B. (2006). Research and Development of an Efficient Process for the Construction of the 2,4,5-Substituted Pyridines of NK-1 Receptor Antagonists. Org. Process Res. Dev..

[58] Fadini L., Manini P., Pietra C., Giuliano C., Lovati E., Cannella R., Venturini A., Stella V.J. (2018). Substituted Piperaziniums for the Treatment of Emesis. U.S. Patent.

[59] Roberts A.W. (2020). Therapeutic development and current uses of BCL-2 inhibition. Hematology.

[60] Yap J.L., Chen L., Lanning M.E., Fletcher S. (2017). Expanding the Cancer Arsenal with Targeted Therapies: Disarmament of the Antiapoptotic Bcl-2 Proteins by Small Molecules. J. Med. Chem..

[61] Souers A.J., Leverson J.D., Boghaert E.R., Ackler S.L., Catron N.D., Chen J., Dayton B.D., Ding H., Enschede S.H., Fairbrother W.J. (2013). ABT-199, a potent and selective BCL-2 inhibitor, achieves antitumor activity while sparing platelets. Nat. Med..

[62] Ku Y.-Y., Chan V.S., Christesen A., Grieme T., Mulhern M., Pu Y.-M., Wendt M.D. (2019). Development of a Convergent Large-Scale Synthesis for Venetoclax, a First-in-Class BCL-2 Selective Inhibitor. J. Org. Chem..

[63] Citrome L. (2015). Brexpiprazole: A new dopamine D2 receptor partial agonist for the treatment of schizophrenia and major depressive disorder. Drugs Today.

[64] Siwek M., Wojtasik-Bakalarz K., Krupa A.J., Chrobak A.A. (2023). Brexpiprazole-Pharmacologic Properties and Use in Schizophrenia and Mood Disorders. Brain Sci..

[65] Yamashita H., Matsubara J., Oshima K., Kuroda H., Ito N., Miyamura S., Shimizu S., Tanaka T., Oshiro Y., Shimada J. (2006). Piperazine-Substituted Benzothiophenes for Treatment of Mental Disorders.

[66] Miyake M., Shimizu M., Tsuji K., Ikeda K. (2016). Safe and Efficient Decarboxylation Process: A Practical Synthetic Route to 4-Chlorobenzo[b]thiophene. Org. Process Res. Dev..

[67] Shinhama K., Utsumi N., Sota M., Fujieda S., Ogasawara S. (2013). Method for Producing benzo[b]thiophene Compound.

[68] Kumar A.S., Kandanur S.G.S., Sen S., Oruganti S. (2018). Delineating an alternate convergent synthesis of brexpiprazole: A novel use of commercial 6,7-dihydrobenzo[b]thiophen-4(5H)-one as precursor to an efficacious Buchwald–Hartwig amination step. J. Chem. Sci..

[69] Wu C., Chen W., Jiang D., Jiang X., Shen J. (2015). An Improved Synthesis of 4-(1-Piperazinyl)benzo[b]thiophene Dihydrochloride. Org. Process Res. Dev..

[70] Heinrich T., Böttcher H., Gericke R., Bartoszyk G.D., Anzali S., Seyfried C.A., Greiner H.E., Van Amsterdam C. (2004). Synthesis and Structure−Activity Relationship in a Class of Indolebutylpiperazines as Dual 5-HT1A Receptor Agonists and Serotonin Reuptake Inhibitors. J. Med. Chem..

[71] Hu B., Song Q., Xu Y. (2012). Scale-Up Synthesis of Antidepressant Drug Vilazodone. Org. Process Res. Dev..

[72] Borsini F., Evans K., Jason K., Rohde F., Alexander B., Pollentier S. (2002). Pharmacology of Flibanserin. CNS Drug Rev..

[73] Dooley E.M., Miller M.K., Clayton A.H. (2017). Flibanserin: From Bench to Bedside. Sex. Med. Rev..

[74] Bietti G., Borsini F., Turconi M., Giraldo E., Bignott M. (1996). Benzimidazolone Derivatives. U.S. Patent.

[75] Yang F., Wu C., Li Z., Tian G., Wu J., Zhu F., Zhang J., He Y., Shen J. (2016). A Facile Route of Synthesis for Making Flibanserin. Org. Process Res. Dev..

[76] Bugaenko D.I., Yurovskaya M.A., Karchava A.V. (2018). N-Arylation of DABCO with Diaryliodonium Salts: General Synthesis of N-Aryl-DABCO Salts as Precursors for 1,4-Disubstituted Piperazines. Org. Lett..

[77] Pahwa M., Sleem A., Elsayed O.H., Good M.E., El-Mallakh R.S. (2021). New Antipsychotic Medications in the Last Decade. Curr. Psychiatry Rep..

[78] Rohde M., M∅Rk N., Håkansson A.E., Jensen K.G., Pedersen H., Dige T., J∅Rgensen E.B., Holm R. (2014). Biological conversion of aripiprazole lauroxil—An N-acyloxymethyl aripiprazole prodrug. Results Pharma Sci..

[79] Ágai-Csongor É., Domány G., Nógrádi K., Galambos J., Vágó I., Keserű G.M., Greiner I., Laszlovszky I., Gere A., Schmidt É. (2012). Discovery of cariprazine (RGH-188): A novel antipsychotic acting on dopamine D3/D2 receptors. Bioorg. Med. Chem. Lett..

[80] Stahl S.M. (2016). Mechanism of action of cariprazine. CNS Spectr..

[81] Oshiro Y., Sato S., Kurahashi N., Tanaka T., Kikuchi T., Tottori K., Uwahodo Y., Nishi T. (1998). Novel Antipsychotic Agents with Dopamine Autoreceptor Agonist Properties:  Synthesis and Pharmacology of 7-[4-(4-Phenyl-1-piperazinyl)butoxy]-3,4-dihydro-2(1H)-quinolinone Derivatives. J. Med. Chem..

[82] Pollard C.B., Wicker T.H. (1954). Derivatives of Piperazine. XXIV. Synthesis of 1-Arylpiperazines and Amino Alcohol Derivatives. J. Am. Chem. Soc..

[83] Perry J.M., Hickey M.B., Remenar J.F., Vandiver J. (2013). Pharmaaceutical Compositions Comprising fatty Acid Esters.

[84] Againe Csongor E., Galambos J., Nogradi K., Vago I., Gyertyan I., Kiss B., Laszlovszky I., Laszy J., Saghy K. (2005). (Thio)carbamoyl-cyclohexane Derivatives as D3/D2 Receptor Antagonists.

[85] Bhosle S.D., Itage S.V., Gangapuram B., Eppa G., Bhosale R.S., Yadav J.S. (2022). Review of Synthetic Approaches toward the Synthesis of Cariprazine, an Antipsychotic Drug. Org. Process Res. Dev..

[86] Againe Csongor E., Czibule L., Seboek F., Juhasz B., Galambos J., Nogradi K. (2010). Process for the Preparation of Piperazine Derivatives.

[87] Czibula L., Againe Csongor E., Nogradi K., Juhasz B., Sebok F., Galambos J., Vago I. (2011). Piperazine Salt and a Process for the Preparation Thereof. U.S. Patent.

[88] Neu J., Garadnay S., Szabó T. (2018). Industrial Process for the Preparation of Cariprazine.

[89] Afanasyev O.I., Kuchuk E., Usanov D.L., Chusov D. (2019). Reductive Amination in the Synthesis of Pharmaceuticals. Chem. Rev..

[90] Magano J., Dunetz J.R. (2012). Large-Scale Carbonyl Reductions in the Pharmaceutical Industry. Org. Process Res. Dev..

[91] Roskoski R. (2022). Targeting BCR-Abl in the treatment of Philadelphia-chromosome positive chronic myelogenous leukemia. Pharmacol. Res..

[92] Yumura M., Nagano T., Nishimura Y. (2020). Novel Multitarget Therapies for Lung Cancer and Respiratory Disease. Molecules.

[93] Boschelli D.H., Ye F., Wang Y.D., Dutia M., Johnson S.L., Wu B., Miller K., Powell D.W., Yaczko D., Young M. (2001). Optimization of 4-Phenylamino-3-quinolinecarbonitriles as Potent Inhibitors of Src Kinase Activity. J. Med. Chem..

[94] Boschelli D.H., Wang Y.D., Johnson S., Wu B., Ye F., Barrios Sosa A.C., Golas J.M., Boschelli F. (2004). 7-Alkoxy-4-phenylamino-3-quinolinecar-bonitriles as Dual Inhibitors of Src and Abl Kinases. J. Med. Chem..

[95] Sutherland K., Feigelson G.B., Boschelli D.H., Blum D.M., Strong H.L. (2005). Process for Preparation of 4-amino-3-quinolinecarbonitriles.

[96] Huang W.-S., Metcalf C.A., Sundaramoorthi R., Wang Y., Zou D., Thomas R.M., Zhu X., Cai L., Wen D., Liu S. (2010). Discovery of 3-[2-(Imidazo[1,2-b]pyridazin-3-yl)ethynyl]-4-methyl-N-{4-[(4-methylpiperazin-1-yl)methyl]-3-(trifluoromethyl)phenyl}benzamide (AP24534), a Potent, Orally Active Pan-Inhibitor of Breakpoint Cluster Region-Abelson (BCR-ABL) Kinase Including the T315I Gatekeeper Mutant. J. Med. Chem..

[97] Roth G.J., Heckel A., Colbatzky F., Handschuh S., Kley J., Lehmann-Lintz T., Lotz R., Tontsch-Grunt U., Walter R., Hilberg F. (2009). Design, Synthesis, and Evaluation of Indolinones as Triple Angiokinase Inhibitors and the Discovery of a Highly Specific 6-Methoxycarbonyl-Substituted Indolinone (BIBF 1120). J. Med. Chem..

[98] Heckel A., Roth G.J., Walter R., Van Meel J., Redemann N., Tontsch-Grunt U., Spevak W., Hilberg F. (2001). 6-Position Substituted Indoline, Production and Use Thereof as a Medicament.

[99] Shirley M. (2021). Maralixibat: First Approval. Drugs.

[100] Banerjee S.C., Huang H.-C., Li J.J., Miller R.E., Reitz D.B., Tremont S.J. (2000). Substituted 5-aryl-benzothiepines Having Activity as Inhibitors of Ileal Bile Acid Transport and Taurocholate Uptake. U.S. Patent.

[101] Mudipalli P.S., Pozzo M.J., Park J.M. (2003). Method for the Preparation of Crystalline Tetrahydrobenzothiepines.

[102] Coates D.A., Gelbert L.M., Knobeloch J.M., De Dios Magana A., De Prado Gonzalez A., Filadelfa Del Prado C.M., Garcia Paredes M.C., Martin De La Nava E.M., Martin Ortega Finger M.D., Martinez Perez J.A. (2010). Protein Kinase Inhibitors. U.S. Patent.

[103] Chan E.M. (2015). Combination Therapy for Cancer.

[104] Frederick M.O., Kjell D.P. (2015). A synthesis of abemaciclib utilizing a Leuckart–Wallach reaction. Tetrahedron Lett..

[105] Mori M., Kaneko N., Ueno Y., Yamada M., Tanaka R., Saito R., Shimada I., Mori K., Kuromitsu S. (2017). Gilteritinib, a FLT3/AXL inhibitor, shows antileukemic activity in mouse models of FLT3 mutated acute myeloid leukemia. Investig. New Drugs.

[106] Huang W.-S., Liu S., Zou D., Thomas M., Wang Y., Zhou T., Romero J., Kohlmann A., Li F., Qi J. (2016). Discovery of Brigatinib (AP26113), a Phosphine Oxide-Containing, Potent, Orally Active Inhibitor of Anaplastic Lymphoma Kinase. J. Med. Chem..

[107] Shimada I., Kurosawa K., Matsuya T., Iikubo K., Kondoh Y., Kamikawa A., Tomiyama H., Iwai Y. (2010). Diamino Heterocyclic Carboxamide Compound. U.S. Patent.

[108] Yue Q., Zhou Z., Gao Q., Baofu Z. (2016). Synthesis Method of 3,5-Disubstituted-pyrazine-2-formamide Compound.

[109] Iikubo K., Kondoh Y., Shimada I., Matsuya T., Mori K., Ueno Y., Okada M. (2018). Discovery of *N*-{2-Methoxy-4-[4-(4-methylpiperazin-1-yl)piperidin-1-yl]phenyl}-*N*′-[2-(propane-2-sulfonyl)phenyl]-1,3,5-triazine-2,4-diamine (ASP3026), a Potent and Selective Anaplastic Lymphoma Kinase (ALK) Inhibitor. Chem. Pharm. Bull..

[110] Högnäsbacka A.A., Poot A.J., Kooijman E., Schuit R.C., Schreurs M., Verlaan M., Beaino W., Van Dongen G.a.M.S., Vugts D.J., Windhorst A.D. (2023). Synthesis and Preclinical Evaluation of [Methylpiperazine-^11^C]brigatinib as a PET Tracer Targeting Both Mutated Epidermal Growth Factor Receptor and Anaplastic Lymphoma Kinase. J. Med. Chem..

[111] Kung C., Hixon J., Choe S., Marks K., Gross S., Murphy E., Delabarre B., Cianchetta G., Sethumadhavan S., Wang X. (2012). Small Molecule Activation of PKM2 in Cancer Cells Induces Serine Auxotrophy. Chem. Biol..

[112] Kung C., Hixon J., Kosinski P.A., Cianchetta G., Histen G., Chen Y., Hill C., Gross S., Si Y., Johnson K. (2017). AG-348 enhances pyruvate kinase activity in red blood cells from patients with pyruvate kinase deficiency. Blood.

[113] Rab M.a.E., Van Oirschot B.A., Kosinski P.A., Hixon J., Johnson K., Chubukov V., Dang L., Pasterkamp G., Van Straaten S., Van Solinge W.W. (2020). AG-348 (Mitapivat), an allosteric activator of red blood cell pyruvate kinase, increases enzymatic activity, protein stability, and ATP levels over a broad range of PKLR genotypes. Haematologica.

[114] Salituro F.G., Saunders J.O., Yan S. (2010). Therapeutic Compounds and Compositions. U.S. Patent.

[115] Sizemore J., Guo L., Mirmehrabi M., Su Y. (2019). Crystalline Forms of n-(4-(4-(cyclopropylmethyl) piperazine-1-carbonyl)phenyl)quinoline-8-sulfonamide.

[116] Dhillon S. (2023). Zavegepant: First Approval. Drugs.

[117] Cann R.O., Chen C.-P.H., Gao Q., Hanson R.L., Hsieh D., Li J., Lin D., Parsons R.L., Pendri Y., Nielsen R.B. (2012). Selection of an Enantioselective Process for the Preparation of a CGRP Receptor Inhibitor. Org. Process Res. Dev..

[118] Chaturvedula P.V., Mercer S.E., Pin S.S., Thalody G., Xu C., Conway C.M., Keavy D., Signor L., Cantor G.H., Mathias N. (2013). Discovery of (R)-N-(3-(7-methyl-1H-indazol-5-yl)-1-(4-(1-methylpiperidin-4-yl)-1-oxopropan-2-yl)-4-(2-oxo-1,2-dihydroquinolin-3-yl)piperidine-1-carboxamide (BMS-742413): A potent human CGRP antagonist with superior safety profile for the treatment of migraine through intranasal delivery. Bioorg. Med. Chem. Lett..

[119] Ryan K., Bolaňos B., Smith M., Palde P.B., Cuenca P.D., Vanarsdale T.L., Niessen S., Zhang L., Behenna D., Ornelas M.A. (2021). Dissecting the molecular determinants of clinical PARP1 inhibitor selectivity for tankyrase1. J. Biol. Chem..

[120] Ferraris D.V. (2010). Evolution of Poly(ADP-ribose) Polymerase-1 (PARP-1) Inhibitors. From Concept to Clinic. J. Med. Chem..

[121] Menear K.A., Adcock C., Boulter R., Cockcroft X.-L., Copsey L., Cranston A., Dillon K.J., Drzewiecki J., Garman S., Gomez S. (2008). 4-[3-(4-Cyclopropanecarbonylpiperazine-1-carbonyl)-4-fluorobenzyl]-2H-phthalazin-1-one: A Novel Bioavailable Inhibitor of Poly(ADP-ribose) Polymerase-1. J. Med. Chem..

[122] Menear K.A., Ottridge A.P., Londesbrough D.J., Hallett M.R., Mullholland K.R., Pittam J.D., Laffan D.D.P., Ashworth I.W., Jones M.F., Cherryman J.H. (2008). Phthalazinone Derivative.

[123] Zmuda F., Malviya G., Blair A., Boyd M., Chalmers A.J., Sutherland A., Pimlott S.L. (2015). Synthesis and Evaluation of a Radioiodinated Tracer with Specificity for Poly(ADP-ribose) Polymerase-1 (PARP-1) in Vivo. J. Med. Chem..

[124] Lai Y.-T. (2021). Small Molecule HIV-1 Attachment Inhibitors: Discovery, Mode of Action and Structural Basis of Inhibition. Viruses.

[125] Meanwell N.A., Krystal M.R., Nowicka-Sans B., Langley D.R., Conlon D.A., Eastgate M.D., Grasela D.M., Timmins P., Wang T., Kadow J.F. (2018). Inhibitors of HIV-1 Attachment: The Discovery and Development of Temsavir and its Prodrug Fostemsavir. J. Med. Chem..

[126] Wang T., Ueda Y., Zhang Z., Yin Z., Matiskella J., Pearce B.C., Yang Z., Zheng M., Parker D.D., Yamanaka G.A. (2018). Discovery of the Human Immunodeficiency Virus Type 1 (HIV-1) Attachment Inhibitor Temsavir and Its Phosphonooxymethyl Prodrug Fostemsavir. J. Med. Chem..

[127] Soundararajan N., Qiu Y., Hu W., Kronenthal D.R., Sirard P., Lajeunesse J., Droghini R., Chidambaram R., Qian X., Natalie K.J. (2006). Process for Preparing Triazole Substituted Azaindoleoxoacetic Piperazine Derivatives and Novel Salt Forms Produced Therein. U.S. Patent.

[128] Saha D., Ryan K.R., Lakkaniga N.R., Acharya B., Garcia N.G., Smith E.L., Frett B. (2021). Targeting Rearranged during Transfection in Cancer: A Perspective on Small-Molecule Inhibitors and Their Clinical Development. J. Med. Chem..

[129] Subbiah V., Shen T., Terzyan S.S., Liu X., Hu X., Patel K.P., Hu M., Cabanillas M., Behrang A., Meric-Bernstam F. (2021). Structural basis of acquired resistance to selpercatinib and pralsetinib mediated by non-gatekeeper RET mutations. Ann. Oncol..

[130] Andrews S.W., Aronow S., Blake J.F., Brandhuber B.J., Cook A., Haas J., Jiang Y., Kolakowski G.R., Mcfaddin E.A., Mckenney M.L. (2018). Substituted Pyrazolo[1,5-a]pyridine Compounds as RET Kinase Inhibitors.

[131] Eary C.T., Spencer S., Crane Z., Chando K., Asselin S., Liu W., Welch M., Cook A., Kolakowski G.R., Metcalf A.T. (2019). Process for the Preparation of 6-(2-Hydroxy-2-methylpropoxy)-4-(6-(6-((6-methoxypyridin-3-yl)methyl)-3,6-diazabicyclo[3.1.1]heptan-3-yl)pyridin-3-yl)pyrazolo[1,5-a]pyridine-3-carbonitrile. Patent.

[132] Ratni H., Ebeling M., Baird J., Bendels S., Bylund J., Chen K.S., Denk N., Feng Z., Green L., Guerard M. (2018). Discovery of Risdiplam, a Selective Survival of Motor Neuron-2 (SMN2) Gene Splicing Modifier for the Treatment of Spinal Muscular Atrophy (SMA). J. Med. Chem..

[133] Campagne S., Boigner S., Rüdisser S., Moursy A., Gillioz L., Knörlein A., Hall J., Ratni H., Cléry A., Allain F.H.T. (2019). Structural basis of a small molecule targeting RNA for a specific splicing correction. Nat. Chem. Biol..

[134] Adam J.-M., Fantasia S.M., Fishlock D.V., Hoffmann-Emery F., Moine G., Pfleger C., Moessner C. (2019). Process for the Prepration of 7-(4,7-Diazaspiro[2.5]octan-7-yl)-2-(2,8-dimethylimidazo[1,2-b]pyridazin-6-yl)pyrido[1,2-a]pyrimidin-4-one Derivatives.

[135] Adam J.-M., Pfleger C., Wuitschik G. (2022). Novel Process.

[136] Crump B.R., Goss C., Lovelace T., Lewis R., Peterson J. (2013). Influence of Reaction Parameters on the First Principles Reaction Rate Modeling of a Platinum and Vanadium Catalyzed Nitro Reduction. Org. Process Res. Dev..

[137] Zhao D., Liu Y., Yi F., Zhao X., Lu K. (2023). Recent advances in the development of inhibitors targeting KRAS-G12C and its related pathways. Eur. J. Med. Chem..

[138] Zhang L., Griffin D.J., Beaver M.G., Blue L.E., Borths C.J., Brown D.B., Caille S., Chen Y., Cherney A.H., Cochran B.M. (2022). Development of a Commercial Manufacturing Process for Sotorasib, a First-in-Class KRASG12C Inhibitor. Org. Process Res. Dev..

[139] Lanman B.A., Allen J.R., Allen J.G., Amegadzie A.K., Ashton K.S., Booker S.K., Chen J.J., Chen N., Frohn M.J., Goodman G. (2020). Discovery of a Covalent Inhibitor of KRASG12C (AMG 510) for the Treatment of Solid Tumors. J. Med. Chem..

[140] Parsons A.T., Caille S., Caporini M.A., Griffin D.J., Lovette M.A., Powazinik W.I.V., St-Pierre G. (2022). Axial Chirality in the Sotorasib Drug Substance, Part 1: Development of a Classical Resolution to Prepare an Atropisomerically Pure Sotorasib Intermediate. Org. Process Res. Dev..

[141] Blake J.F., Burgess L.E., Chicarelli M.J., Christensen J.G., Cook A., Fell J.B., Fischer J.P., Marx M.A., Mejia M.J., Savechenkov P. (2019). KRAS G12C Inhibitors. U.S. Patent.

[142] Fell J.B., Fischer J.P., Baer B.R., Blake J.F., Bouhana K., Briere D.M., Brown K.D., Burgess L.E., Burns A.C., Burkard M.R. (2020). Identification of the Clinical Development Candidate MRTX849, a Covalent KRASG12C Inhibitor for the Treatment of Cancer. J. Med. Chem..

[143] Snead D.R., Gan Y., Scattolin T., Paymode D.J., Achmatowicz M., Rudisill D.E., Vidal E.S., Gharbaoui T., Roberts P., Yang J. (2023). Development of Adagrasib’s Commercial Manufacturing Route. Org. Process Res. Dev..

[144] Chen C.-Y., Lu Z., Scattolin T., Chen C., Gan Y., Mclaughlin M. (2023). Synthesis of Adagrasib (MRTX849), a Covalent KRASG12C Inhibitor Drug for the Treatment of Cancer. Org. Lett..

[145] Lee A. (2023). Fezolinetant: First Approval. Drugs.

[146] Hoveyda H.R., Fraser G.L., Roy M.-O., Dutheuil G., Batt F., El Bousmaqui M., Korac J., Lenoir F., Lapin A., Noël S. (2015). Discovery and Optimization of Novel Antagonists to the Human Neurokinin-3 Receptor for the Treatment of Sex-Hormone Disorders (Part I). J. Med. Chem..

[147] Hoveyda H.R., Fraser G.L., Dutheuil G., El Bousmaqui M., Korac J., Lenoir F., Lapin A., Noël S. (2015). Optimization of Novel Antagonists to the Neurokinin-3 Receptor for the Treatment of Sex-Hormone Disorders (Part II). ACS Med. Chem. Lett..

[148] Hoveyda H.R., Dutheuil G. (2016). Novel Chiral Synthesis of N-Acyl-(3-substituted)-(8-substituted)-5,6-dihydro-[1,2,4]triazolo[4,3-a]pyrazines.

[149] Blair H.A. (2020). Lumateperone: First Approval. Drugs.

[150] Li P., Zhang Q., Robichaud A.J., Lee T., Tomesch J., Yao W., Beard J.D., Snyder G.L., Zhu H., Peng Y. (2014). Discovery of a Tetracyclic Quinoxaline Derivative as a Potent and Orally Active Multifunctional Drug Candidate for the Treatment of Neuropsychiatric and Neurological Disorders. J. Med. Chem..

[151] Li P., Zhang Q. (2019). Substituted Heterocycle Fused Gamma-Carbolines Synthesis.

[152] Kang C. (2021). Fosdenopterin: First Approval. Drugs.

[153] Clinch K., Watt D.K., Dixon R.A., Baars S.M., Gainsford G.J., Tiwari A., Schwarz G., Saotome Y., Storek M., Belaidi A.A. (2013). Synthesis of Cyclic Pyranopterin Monophosphate, a Biosynthetic Intermediate in the Molybdenum Cofactor Pathway. J. Med. Chem..

[154] De Sanctis R., Jacobs F., Benvenuti C., Gaudio M., Franceschini R., Tancredi R., Pedrazzoli P., Santoro A., Zambelli A. (2022). From seaside to bedside: Current evidence and future perspectives in the treatment of breast cancer using marine compounds. Front. Pharmacol..

[155] Pommier Y., Kohlhagen G., Bailly C., Waring M., Mazumder A., Kohn K.W. (1996). DNA Sequence- and Structure-Selective Alkylation of Guanine N2 in the DNA Minor Groove by Ecteinascidin 743, a Potent Antitumor Compound from the Caribbean Tunicate Ecteinascidia turbinata. Biochemistry.

[156] He W., Zhang Z., Ma D. (2019). A Scalable Total Synthesis of the Antitumor Agents Et-743 and Lurbinectedin. Angew. Chem. Int. Ed..

[157] Martin Lopez M.J., Francesch Solloso A., Cuevas Marchante M.D.C. (2011). Synthetic Process for the Manufacture of Ecteinascidin Compounds.

[158] Corey E.J., Gin D.Y., Kania R.S. (1996). Enantioselective Total Synthesis of Ecteinascidin 743. J. Am. Chem. Soc..

[159] Johns B.A., Kawasuji T., Weatherhead J.G., Taishi T., Temelkoff D.P., Yoshida H., Akiyama T., Taoda Y., Murai H., Kiyama R. (2013). Carbamoyl Pyridone HIV-1 Integrase Inhibitors 3. A Diastereomeric Approach to Chiral Nonracemic Tricyclic Ring Systems and the Discovery of Dolutegravir (S/GSK1349572) and (S/GSK1265744). J. Med. Chem..

[160] Zhao A.V., Crutchley R.D., Guduru R.C., Ton K., Lam T., Min A.C. (2022). A clinical review of HIV integrase strand transfer inhibitors (INSTIs) for the prevention and treatment of HIV-1 infection. Retrovirology.

[161] Johns B.A., Kawasuji T., Taishi T., Taoda Y. (2006). Polycyclic Carbamoylpyridone Derivative Having Hiv Integrase Inhibitory Activity.

[162] Jin H., Lazerwith S.E., Martin T.A.T., Bacon E.M., Cottell J.J., Cai Z.R., Pyun H.-J., Morganelli P.A., Ji M., Taylor J.G. (2014). Polycyclic-Carbamoylpyridone Compounds and Their Pharmaceutical Use.

[163] Huang H.-C., Tremont S.J., Lee L.F., Keller B.T., Carpenter A.J., Wang C.-C., Banerjee S.C., Both S.R., Fletcher T., Garland D.J. (2005). Discovery of Potent, Nonsystemic Apical Sodium-Codependent Bile Acid Transporter Inhibitors (Part 2). J. Med. Chem..

[164] Wendt M.D., Shen W., Kunzer A., Mcclellan W.J., Bruncko M., Oost T.K., Ding H., Joseph M.K., Zhang H., Nimmer P.M. (2006). Discovery and Structure−Activity Relationship of Antagonists of B-Cell Lymphoma 2 Family Proteins with Chemopotentiation Activity in Vitro and in Vivo. J. Med. Chem..

[165] Soskic V., Sukalovic V., Kostic-Rajacic S. (2015). Exploration of N-arylpiperazine Binding Sites of D2 Dopaminergic Receptor. Mini-Rev. Med. Chem..

[166] Möller D., Salama I., Kling R.C., Hübner H., Gmeiner P. (2015). 1,4-Disubstituted aromatic piperazines with high 5-HT2A/D2 selectivity: Quantitative structure-selectivity investigations, docking, synthesis and biological evaluation. Bioorg. Med. Chem..

[167] Partyka A., Kurczab R., Canale V., Satała G., Marciniec K., Pasierb A., Jastrzębska-Więsek M., Pawłowski M., Wesołowska A., Bojarski A.J. (2017). The impact of the halogen bonding on D2 and 5-HT1A/5-HT7 receptor activity of azinesulfonamides of 4-[(2-ethyl)piperidinyl-1-yl]phenylpiperazines with antipsychotic and antidepressant properties. Bioorg. Med. Chem..

[168] Ostrem J.M., Peters U., Sos M.L., Wells J.A., Shokat K.M. (2013). K-Ras(G12C) inhibitors allosterically control GTP affinity and effector interactions. Nature.

